# *Oryza*-Specific Orphan Protein Triggers Enhanced Resistance to *Xanthomonas oryzae* pv. *oryzae* in Rice

**DOI:** 10.3389/fpls.2022.859375

**Published:** 2022-03-10

**Authors:** Hyeran Moon, A-Ram Jeong, Oh-Kyu Kwon, Chang-Jin Park

**Affiliations:** ^1^Department of Molecular Biology, Sejong University, Seoul, South Korea; ^2^Department of Bioresources Engineering, Sejong University, Seoul, South Korea

**Keywords:** Arabidopsis, *Pseudomonas syringae* pv. *tomato* DC3000, rice, taxonomically restricted genes, lineage-specific gene, *Xanthomonas oryzae* pv. *oryzae*, orphan

## Abstract

All genomes carry lineage-specific orphan genes lacking homology in their closely related species. Identification and functional study of the orphan genes is fundamentally important for understanding lineage-specific adaptations including acquirement of resistance to pathogens. However, most orphan genes are of unknown function due to the difficulties in studying them using helpful comparative genomics. Here, we present a defense-related *Oryza*-specific orphan gene, *Xio1*, specifically induced by the bacterial pathogen *Xanthomonas oryzae* pv. *oryzae* (*Xoo*) in an immune receptor XA21-dependent manner. Salicylic acid (SA) and ethephon (ET) also induced its expression, but methyl jasmonic acid (MeJA) reduced its basal expression. C-terminal green fluorescent protein (GFP) tagged Xio1 (Xio1-GFP) was visualized in the nucleus and the cytosol after polyethylene glycol (PEG)-mediated transformation in rice protoplasts and Agrobacterium-mediated infiltration in tobacco leaves. Transgenic rice plants overexpressing Xio1-GFP showed significantly enhanced resistance to *Xoo* with reduced lesion lengths and bacterial growth, in company with constitutive expression of defense-related genes. However, all of the transgenic plants displayed severe growth retardation and premature death. Reactive oxygen species (ROS) was significantly produced in rice protoplasts constitutively expressing Xio1-GFP. Overexpression of Xio1-GFP in non-*Oryza* plant species, *Arabidopsis thaliana*, failed to induce growth retardation and enhanced resistance to *Pseudomonas syringae* pv. *tomato* (*Pst*) DC3000. Our results suggest that the defense-related orphan gene *Xio1* plays an important role in distinctive mechanisms evolved within the *Oryza* and provides a new source of *Oryza*-specific genes for crop-breeding programs.

## Introduction

Orphan genes are protein-coding regions that lack detectable similarity to genes in distantly related species (Tautz and Domazet-Loso, 2011; Arendsee et al., 2014). They are also known as lineage-specific genes, taxonomically restricted genes, species-specific genes, and *de novo* originated new genes (Xiao et al., 2009; Yang et al., 2009; Tautz and Domazet-Loso, 2011). Identification and functional study of the orphan genes is fundamentally important for understanding the origin of new species, species-specific morphological features, and evolution of immune systems (Khalturin et al., 2009; Chen et al., 2013). However, in-depth study of lineage-specific orphan genes is challenging because it requires high-quality reference genomes for closely related species in a genus (Zhang et al., 2019). The genus *Oryza* has 27 species, representing 11 genome types, 6 of which are diploid (*n* = 12: AA, BB, CC, EE, FF, and GG) and 5 of which are polyploid (*n* = 24: BBCC, CCDD, HHJJ, HHKK, and KKLL) (Stein et al., 2018). Domesticated rice species including *O. sativa* ssp. *japonica*, *O. sativa* ssp. *indica*, and *O. glaberrima* belong to the AA genome type, the primary genetic resources in rice improvement. Including the domesticated rice species, the genomes of 13 closely related species have sequenced (Stein et al., 2018), and the genus *Oryza* is now considered as a model system for the identification and study of orphan genes (Zhang et al., 2019).

The proportions and numbers of orphan genes in different species are different from each other and vary considerably even in the same species depending on analytical methods. For example, using bioinformatic computation and statistical analysis, only 1,926 out of 18,398 of total orphan gene candidates in the BLAST result were confirmed as orphan proteins in rice (Guo et al., 2007). However, after comparison of eight representative genomes of green plants, the numbers of genes within orphan gene families from Arabidopsis (*Arabidopsis thaliana*) and rice (*Oryzae sativa*) were predicted as 1,430 and 16,045, respectively (Guo, 2013). It has been believed that orphan genes are important for the survival of organisms under new environmental conditions (Arendsee et al., 2014; Li et al., 2015). Therefore, many of the orphan genes are often enriched in differentially expressed gene sets in response to hormones, abiotic, or biotic stresses, suggesting that at least some of orphan genes should play an important role in signal transduction (Donoghue et al., 2011; Guo, 2013; Arendsee et al., 2014; Perochon et al., 2015). However, the importance of orphan genes has been underappreciated because of difficulties in studying genes where comparative genomics is impossible as well as a bias toward broadly conserved genes (Arendsee et al., 2014). Therefore, there are only a few *in vivo* functional studies of orphan genes related to disease resistance. For example, a Brassicaceae-specific Arabidopsis gene, enhancer of vascular wilt resistance 1 (EWR1), confers resistance to vascular wilt pathogens, *Verticillium* spp. and *Ralstonia solanacearum* (Yadeta et al., 2014), and *Triticum aestivum Fusarium* resistance orphan gene (*TaFROG*) from wheat enhances resistance to the fungal pathogen *Fusarium graminearum* (Perochon et al., 2015). Rice resistance gene, Xa7, against bacterial pathogen *Xanthomonas oryzae* pv. *oryzae* (*Xoo*) causing bacterial blight disease was recently cloned as a new orphan gene (Wang et al., 2021). On the contrary, another rice orphan *Oryza sativa* defense-responsive gene 10 (OsDR10) has a negative effect on the resistance to bacterial blight disease (Xiao et al., 2009).

The XA21, a representative immune receptor, confers broad-spectrum immunity to *Xoo* (Song et al., 1995). A tyrosine-sulfated microbial peptide from *Xoo*, called RaxX-sY (required for activation of XA21-mediated immunity X, tyrosine-sulfated), is recognized by XA21 and triggers XA21-mediated immune responses (Pruitt et al., 2015). Here, we pursued functional characterization of a novel *Oryza*-specific gene, encoding an orphan protein of unknown function. The orphan gene was specifically induced by *Xoo* in an XA21-dependent manner. This protein is designated as *Oryza*-specific *Xoo*-induced orphan 1 (Xio1). Transgenic rice overexpressing *Xio1* displayed enhanced resistance to *Xoo* but led to a severe restriction of plant growth. These data provide strong evidence of its functionality in stress response induced by the bacterial pathogen *Xoo*, suggesting that underappreciated orphan genes should be considered as new sources of defense-related genes for crop-breeding programs.

## Materials and Methods

### Plant Materials and Growth Conditions

Rice (*Oryza sativa* L.) japonica variety “Kitaake” and transgenic Kitaake plants were grown at 28°C during the day and at 20°C at night with a light/dark cycle of 14/10 h (h) in a greenhouse facility at Sejong University in Korea. To grow rice plants in soil, rice seeds sterilized by heating at 65°C for 8 min were incubated in sterile water at 23°C for 7 days and then transferred to Baroker bed soil (Seoul Bio). Fully expanded leaves from 7-week-old rice plants were used for all experiments, unless otherwise indicated.

*Arabidopsis thaliana* ecotype Colombia-0 (Col-0) and transgenic Col-0 were grown on Sungro propagation mix soil (Sun Gro Horticulture) in a growth chamber under 16/8 h light/dark conditions at 23°C and 70% relative humidity. For stratification, Arabidopsis seeds sterilized with 70% ethanol and 20% sodium hypochlorite solution were incubated in sterile water at 4°C for 7 days.

*Nicotiana benthamiana* (tobacco) were maintained in Baroker potting soil (Seoul Bio)_in a growth chamber under 16/8 h light/dark conditions at 23°C and 70% relative humidity. Tobacco seeds sterilized with 70% ethanol and 40% sodium hypochlorite solution were germinated in Murashige and Skoog (MS) medium (Murashige and Skoog, 1962) at 23°C for 7 days and then transferred to soil.

### Xio1 Homologous Collection

Sequences of Xio1 homologous were downloaded from the entire set of available species in the Ensembl Plants database^1^ (Zerbino et al., 2018). Of the 96 plant species occurring in the database, 11 belong to the genus *Oryza*. Xio1 protein sequence (LOC_Os09g13440.1) was used as a query against the available downloaded plant proteomes using BLASTp strategy (*E* value < 10^–6^) and then against the available downloaded plant genomes using tBLASTn (*E* value < 10^–10^). Afterward, multiple sequence alignments of protein sequences of Xio1 homologous were performed using the Mega7 software.

### Vector Construction

To generate a construct overexpressing Xio1 tagged with green fluorescent protein (Xio1-GFP) at its C-terminus in rice, full-length complementary DNA (cDNA) of *Xio1* with a removed stop codon was amplified from the Kitaake cDNA library by the polymerase chain reaction (PCR) using *Xio1*-specific primers, CACC_09g13440_FL_F (5′- CACCATGTCGAAATGCACAGCAAATA-3′) and 09g13440_ FL_woSTOP_R (5′-TGCTGGGTTTCTTCTGCGCCC-3′). The PCR amplified product was cloned into pENTR/GW/TOPO^®^ vector (Invitrogen) to create an Xio1_woSTOP/pENTR. The overexpression construct, Ubi:Xio1-GFP/pC1300, was obtained by recombining the Xio1_woSTOP/pENTR construct into the gateway vectors Ubi:GFP/CAMBIA1300 (Rohila et al., 2006; Park et al., 2017) through a Gateway^®^ LR clonase^®^ II reaction (Invitrogen). The construct was also used for subcellular localization study in rice protoplasts.

As a positive control for accumulating ROS, full-length cDNA of rice salt intolerance 1 (*OsSIT1*) (Li et al., 2014), was amplified using *OsSIT1*-specific primers, CACC_OsSIT1_Short_FL_F (5′-CACCATGCTGACGAACGGCACGA-3′) and OsSIT1_FL_woS TOP_R (5′-TGCTCTCGCTCGAGGAATGTCA-3′). The final construct, Ubi:OsSIT1-GFP/pC1300, was obtained by recombining the OsSIT1_woSTOP/pENTR construct into the gateway vectors Ubi:GFP/CAMBIA1300 (Rohila et al., 2006; Park et al., 2017) through a Gateway^®^ LR clonase^®^ II reaction (Invitrogen).

To generate a construct overexpressing Xio1-GFP in Arabidopsis Col-0 and tobacco plants, the 35S:Xio1-GFP/pEG103 construct was generated by recombining the Xio1_woSTOP/pENTR construct into the gateway vector pEarleyGate 103 (pEG103) (Earley et al., 2006) through a Gateway^®^ LR clonase^®^ II reaction (Invitrogen).

### Subcellular Localization

Rice protoplasts were harvested from 2-week-old rice seedlings using a previously described method (Bart et al., 2006) with minor modification. Fusion construct (NLS-RFP) of the nuclear localization signal (NLS) from simian virus 40 large T antigen and red fluorescent protein (RFP) was used for artificial localization of RFP into the nucleus (Lee et al., 2001). The vectors, 326-GFP and 326-RFP, were used as free GFP and RFP control, respectively (Lee et al., 2001). Ubi:Xio1-GFP/pC1300 and control plasmids were transformed into rice protoplasts through polyethylene glycol (PEG)-mediated transformation as previously described (Bart et al., 2006). For transient expression in tobacco leaves, *Agrobacterium tumefaciens* strain GV3101 was transformed with the plasmid 35S:Xio1-GFP/pEG103 or control plasmid 35S:YFP/pEG104 (Earley et al., 2006). *A. tumefaciens* cells were cultured, harvested, and resuspended in an infiltration buffer [10 mM MES (Biobasic), 10 mM MgCl_2_ (Biobasic), and 100 μM acetosyringone (Sigma)] (pH 5.7) at a final OD600 = 0.4. Samples were infiltrated into 4 to 5-week-old tobacco leaves with 1-ml syringe. Fluorescences in protoplasts and leaves were photographed using an Eclipse Ti fluorescence microscope (Nikon) fitted with an objective (200× or 400×). The filter sets used were C-FL-C FITC (excitation 465 to 495 nm) and C-FL-C TRITC (excitation 537 to 552 nm) (Nikon) to detect GFP/YFP and RFP, respectively.

### Generation of Transgenic Plants and Transformant Selection

Rice transformations were carried out as described previously (Chern et al., 2005). For PCR selection of transformants carrying the Ubi:Xio1-GFP/pC1300 construct, hygromycin phosphotransferase (*hpt*)-specific primers, HygB_For (5′-CGC AAGGAATCGGTCAATACA-3′) and HygB_Rev (5′-CTACAC AGCCATCGGTCCAGA-3′) or *Xio1-GFP*-specific primers, 09g13440si_292-311_F (5′-GATGTTGTTCTTGGCCAGGG-3′) and smGFP2_308-327_R (5′-ACGTGTCTTGTAGTTCCCGT-3′) were used.

For the construction of transgenic Arabidopsis plants, the 35S:Xio1-GFP/pEG103 and 35S:YFP/pEG104 constructs were transformed into Arabidopsis Col-0 using the floral dip method (Clough and Bent, 1998) mediated by the *Agrobacterium tumefaciens* strain GV3101. Transgenic Arabidopsis Col-0 seeds were plated in selective MS medium containing 10 mg/L glufosinate-ammonium (Sigma) for 10 days and then transferred to soil and grown in growth chamber. For PCR selection, *Xio1-GFP*-specific primers, 09g13440_336-355_F (5′-GTTGGAGGAGATACACAAGG-3′) and smGFP2_308-327_R (5′-ACGTGTCTTGTAGTTCCCGT-3′) and *YFP* primers, EG FP_For (5′-CTACCCCGACCACATGAAG-3′) and smGFP2_ 308-327_R (5′-ACGTGTCTTGTAGTTCCCGT-3′) were used.

### Pathogen Inoculations

*Xanthomonas oryzae* pv. *oryzae* (*Xoo*) Philippine race 6 (PR6) strain PXO99 (referred to as *Xoo* throughout text) was used to inoculate Kitaake. *Xoo* was grown on a peptone sucrose agar (PSA) plate [10 g/L peptone (Duchefa), 10 g/L sucrose (Duchefa), 1 g/L glutamic acid (Duchefa), 16 g/L agar (Biobasic) and pH 7.0] containing 20 mg/L cephalexin (Duchefa) for 3 days. Two leaves of each tiller were inoculated by *Xoo* suspensions (OD600 = 0.5) by the scissors-dip method (Song et al., 1995; Chern et al., 2005). Lesion lengths were measured at the indicated days after inoculation. To harvest *Xoo* for bacterial colony counts, the entire leaves inoculated by the scissors-dip method were harvested and cut into approximately 1 mm pieces with sterile scissors and incubated in 10 mL sterile water for 1 h. After a series of gradient dilutions, the extract was plated on PSA plates containing 20 mg/L of cephalexin (Duchefa). The colonies of each plate were counted after incubation at 37°C for 2 days.

To inoculate Arabidopsis, *Pseudomonas syringae* pv. *tomato* (*Pst*) strain DC3000 was cultured in King’s B (KB) broth [20 g/L peptone (Duchefa), 10 ml/L glycerol (Biobasic), 1 g/L K_2_HPO_4_ (Biobasic), 1.5 g/L agar (Biobasic), 1.5 g/L MgSO_4_ (Biobasic), and pH 7.0 containing 30 mg/ml rifampicin (Duchefa) and 50 mg/L kanamycin (Duchefa)] at 28°C. Bacterial cells harvested by centrifugation were re-suspended in 10 mM MgCl_2_ (Biobasic) solution to OD600 = 0.002. The inoculation was performed by a syringe infiltration method (Liu et al., 2015). Bacterial cell suspension was infiltrated into rosette leaves using 1-ml syringes without needle, and the inoculated plants were maintained in sealed containers to facilitate disease development under high humidity. For quantification of bacterial growth in Arabidopsis, five leaf discs from inoculated leaves were collected at indicated time points and homogenized in sterile water. After a series of gradient dilutions, the homogenates were plated on KB plates containing 15 mg/L agar (Biobasic), 30 mg/L rifampicin (Duchefa) and 50 mg/L kanamycin (Duchefa), and bacterial colonies were counted at 1 day after incubation at 28°c.

### Western Blot Analysis

For Western blot analyses, total proteins were extracted from rice or Arabidopsis plants using a protein extraction buffer [6.8 mM Na_2_HPO_4_ (Biobasic), 3.2 mM NaH_2_PO_4_ (Biobasic), 150 mM NaCl (Biobasic), 2 mM EDTA (Biobasic), 200 mM NaF (Biobasic), 1% Triton X-100 (Biobasic), and 1 mM PMSF (Biobasic)]. Protein concentration was determined using a previously described method (Bradford, 1976). Proteins (100 μg) were loaded per well, separated by 10% sodium dodecyl sulfate polyacrylamide gel electrophoresis (SDS-PAGE), and subsequently blotted onto nitrocellulose membrane (Bio-Rad) for immunodetection. For Xio1-GFP detection, anti-GFP antibody (B-2) SC-9996 (Santa Cruz Biotechnology) and horseradish peroxidase (HRP)-conjugated anti-mouse IgG antibody SC-2031, (Santa Cruz Biotechnology) were used as a primary and a secondary antibody at a final dilution of 1:1,000 and 1:10,000 for 2 h, respectively. GFP bands were visualized using the SuperSignal West Pico Chemiluminescent Substrate (Thermo).

### Expression Analysis

For hormone and peptide treatments, detached leaf bioassays were conducted. Rice leaf sections (1.5 to 2.0 cm) were prepared from fully expanded leaves of Kitaake plants. The leaf tip and basal portion were discarded. Leaf sections were equilibrated overnight in 6-well cell culture plates (SPL Life Sciences) filled with sterile water under constitutive light. Then, 200 μM salicylic acid, 1 mM ethephon, or 100 μM methyl jasmonate were substituted with sterile water. A synthetic 21-amino acid derivative of tyrosine-sulfated peptide called RaxX-sY and inactive non-sulfated RaxX (RaxX) were synthesized (Anygene) and treated as described previously (Pruitt et al., 2015; Thomas et al., 2016). For *Xoo* treatment, smear inoculation method was carried out as described previously (Nozue et al., 2011). Three layers of gauze were soaked with *Xoo* suspension (OD600 = 0.8) and smeared onto leaves five times. To extract RNA, leaf sections were collected immediately in the indicated time points, frozen in liquid nitrogen, and stored in a deep freezer at 80°C.

Total RNA was extracted by TRIzol reagent (Invitrogen) and two micrograms of total RNA were reverse-transcribed using M-MLV reverse transcriptase (Promega) according to the manufacturers instructions. Expression of *Xio1* and *Xio1-GFP* were confirmed by RT-PCR using 09g13440_F (5′-GCAAGA ACTACCTCGCCGTGGAC-3′)/09g13440_R (5′-CCGTGGGGA GCTGTTCTTGATAG-3′) and 09g13440_F/smGFP2_308- 327_R (5′-ACGTGTCTTGTAGTTCCCGT-3′), respectively. Expression of each gene was determined by PCR using a specific set of primers: *pathogenesis-related protein 3* (*PR3*), PR3-1 (5′-CTTGGACTGCTACAACCAGA-3′) and PR3-2 (5′-CATTGTGGGCATTACTGATG-3′); *PR10*, PR10_RT_F (5′-CGCAGCTCACATTATCAAGTCAGA-3′) and PR10_RT_R (5′-GAAGCAGCAATACGGAGATGGATG-3′); *lipoxygenases* (*LOX*), LOX_F (5′-TGTACGTGCCGAGGGACGAG-3′) and LOX_R (5′-GCGAGCGTCTCCCTCGCGAACTC-3′); *ascorbate peroxidase 8* (*APX8*), APX8-5′ (5′-TGGTCTGATGACCTC CTCTGA-3′) and APX8-3′ (5′-CATGAGCCATGACAACTAGA-3′); rice translation elongation factor 1 alpha (*rEF1*α), rEF1α1048F (5′-ACTGCCACACCTCCCACATTG-3′) and rEF1α1552R (5′-CAACAGTCGAAGGGCAATAATAAGTC-3′). Quantification of RT-PCR bands on raw gel images in the expression analysis was performed in ImageJ by drawing a rectangular region-of-interest within each band and calculating the gray value of each gene relative to one of *rEF1*α.

### Chemiluminescence Measurement of Reactive Oxygen Species (ROS) Production

After isolation of rice protoplasts, Ubi:Xio1-GFP/pC1300 was transformed into the protoplasts. Ubi:OsSIT1-GFP/pC1300 and Ubi:GFP/pC1300 were used as a positive and a negative control, respectively. ROS production was measured in the transformed rice protoplasts using the luminol-based chemiluminescence assay described previously with minor modifications (Murphy and Huerta, 1990; Tiew et al., 2015). After transformation with each construct, 1 × 10^5^ protoplasts were suspended in each well of a 96-well plate and incubated at RT for approximately 16 h in the dark. 2 × luminol solution (5 mM luminol L-012, 0.25 mg/mL HRP) was added to each well, and luminol-dependent chemiluminescence was measured for 3 h with a signal integration time of 10 s on a luminometer (Berthold Mithras LB940).

### Accession Numbers

Each sequence in this study can be found in the GenBank/EMBL database under following accession numbers: Xio1 (LOC_Os09g13440), Xio1 homologous [*O*. *sativa* ssp. *indica* (B8BE71), *O*. *rufipogon* (A0A0E0QPF5), *O*. *meridionalis* (A0A0E0EQK1), *O*. *glumipatula* (A0A0E0B139), *O*. *barthii* (A0A0D3H4Y5)], OsUDT1 (LOC_Os07g36460), PR3 (LOC_Os06g51060), PR10 (LOC_Os12g36380), LOX (LOC_Os08g39850), APX8 (LOC_Os02g34810), rEF1α (LOC_Os03g08020), OsSIT1 (LOC_Os02g42780), AtPR1 (AT2G14610), AtPR4 (AT3G04720), AtEIF4A1 (AT3G13920).

### Statistical Analysis

All assays described above were repeated at least three times. All data are presented as means ± standard error. Data was analyzed by a one-way analysis of variance (ANOVA) using Sigma Plot 11.0 (Systat Software Inc). Means with different letters in the figures indicate significantly different values at *p* < 0.05 or 0.001 according to the Tukey test.

## Results

### *Xio1* Is an Orphan Gene, Taxonomically Restricted to the Genus *Oryza*

**FIGURE 1:**
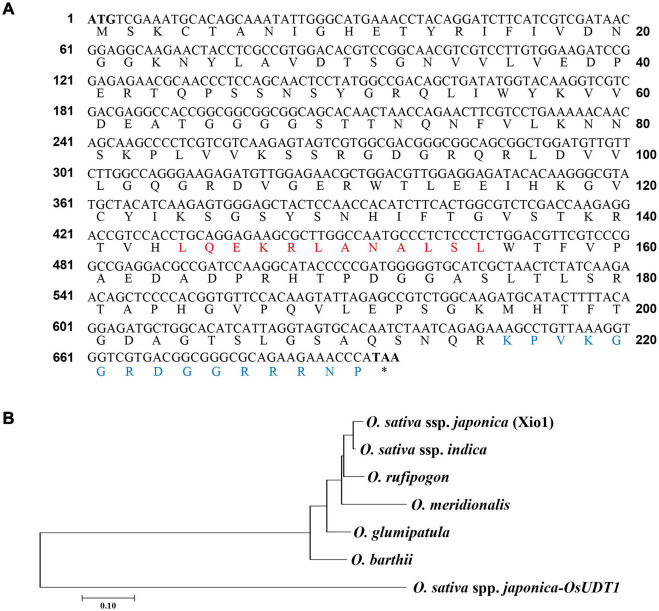
Xio1 is taxonomically restricted to the genus *Oryza*. **(A)** Full-length coding sequence and deduced amino acid sequence of *Xio1*. The start codon (M) and stop codon (asterisks) are in bold font. Red and blue letters represent the conserved position of predicted nuclear export signal (NES) and nuclear localization signal (NLS), respectively. Asterisk (*) indicates a termination codon. **(B)** Phylogenetic analysis of Xio1 and its homologs in genus *Oryza*. The phylogenetic tree of deduced amino acid sequences was generated with the neighbor-joining method using MEGA7 software. The bootstrap consensus tree inferred from 1,000 replicates is taken to represent the evolutionary history of the taxa analyzed. Branches corresponding to partitions reproduced in less than 50% bootstrap replicates are collapsed. OsUDT1 from *O. sativa* subsp. *japonica* was used as outgroup. *Oryza sativa* ssp. *japonica*, Os09g13440; *Oryza glumipatula*, OGLUM09G05440; *Oryza rufipogon*, ORUFI09G05240; *Oryza barthii*, OBART09G04680; *Oryza meridionalis*, OMERI09G03830; *Oryza sativa* ssp. *indica*, BGIOSGA030029.

Previously, we identified XA21-mediated defense-related genes induced by *Xoo*. One of them was predicted to encode an uncharacterized protein without any known domain^2^ (Figure 1A) and cloned for further characterization. To identify its potential homologous using sequence similarity, we ran BLASTp and tBLASTn against the whole plant genome database from Ensembl Plants (see text footnote 1) (Supplementary Table 1). This strategy revealed that its potential homologs are present only in the species within genus *Oryza* (Figure 1B and Supplementary Figure 1). They were restricted to exclusively AA genomes species in the genus *Oryza*, and all hits were mapped to chromosome 9. No variants were identified outside of the genus in any other sequenced monocotyledon and dicotyledon genomes. Therefore, the putative uncharacterized protein was concluded to be a taxonomically restricted *Oryza*-specific orphan protein and designated as *Oryza*-specific *Xoo*-induced orphan 1 (Xio1).

### *Xio1* Is Induced by *Xoo* and Sulfated Microbial Peptide RaxX-sY in an XA21-Dependent Manner

**FIGURE 2:**
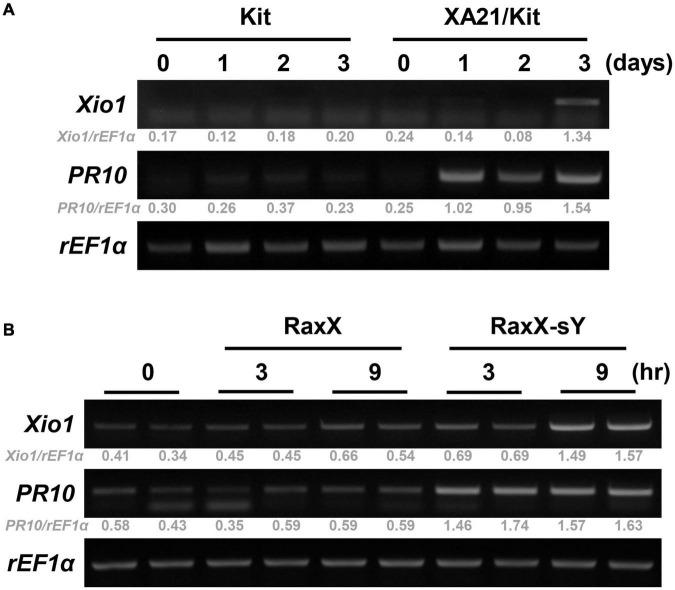
*Xio1* is specifically induced by *Xanthomonas oryzae* pv. *oryzae* and sulfated microbial peptide RaxX in an XA21-dependent manner. **(A)** Expression of *Xio1* gene in Kitaake (Kit) and transgenic Kitaake overexpressing XA21 (XA21/Kit) after *Xoo* inoculation. Fully expanded leaves of 10-week-old rice plants were inoculated with *Xoo* using smear inoculation method and harvested at 0, 1, 2, and 3 days after inoculation. RT-PCR was performed with *Xio1*-specific primers using total RNA extracted from *Xoo*-inoculated leaves from Kitaake and XA21/Kit plants. Pathogenesis-related protein 10 (*PR10*) and rice translation elongation factor 1 alpha (*rEF1*α) were used as a positive control for *Xoo* inoculation and an internal control, respectively. **(B)** Expression of *Xio1* gene in XA21/Kit plants after treatments with synthetic 21-amino acid sulfated derivative of RaxX peptide (RaxX-sY) or inactive non-sulfated peptide (RaxX). In detached leaf bioassay, leaf sections (1.5 to 2 cm) were floated on 1 μM RaxX-sY or RaxX peptide solution in a cell culture plate and harvested at 3 and 9 h after treatments. *PR10* and *rEF1*α were used as a positive control for sulfated peptide RaxX-sY treatment and an internal control, respectively. Experiments were repeated more than three times, with similar results. Relative gray value of RT-PCR bands was quantified by ImageJ.

To confirm XA21-dependent induction of *Xio1* upon *Xoo* inoculation, its expression pattern was monitored in Kitaake lacking XA21 and transgenic Kitaake carrying XA21 driven by the maize ubiquitin (Ubi) promoter in the Kitaake background (XA21/Kit) (Park et al., 2010; Figure 2A). In RT-PCR analysis, *Xio1* in Kitaake was barely detectable at all time points after *Xoo* inoculation. In contrast to the result in Kitaake, *Xio1* in XA21/Kit was significantly induced at 3 days after *Xoo* inoculation. As a positive control for *Xoo* inoculation, the expression of the rice pathogenesis-related protein 10 gene (*PR10*) (McGee et al., 2001) was examined. Strong induction of the *PR10* was observed at 1 day after *Xoo* inoculation and maintained up to 3 days in XA21/Kit only.

A tyrosine-sulfated peptide called RaxX-sY (required for activation of XA21-mediated immunity X, tyrosine-sulfated) secreted from *Xoo* is sufficient to activate XA21-mediated immune responses (Pruitt et al., 2015). Therefore, to assess whether *Xio1* is specifically induced after XA21 recognizes the sulfated peptides, leaf discs prepared from XA21/Kit were treated with a synthetic 21-amino acid derivative of RaxX-sY or inactive non-sulfated RaxX (Figure 2B). Strong induction of *PR10* gene was observed at 3 and 9 h after RaxX-sY treatment but not after RaxX treatment, indicating that sulfated peptide successfully triggered the XA21-mediated immune response in XA21/Kit. Transcripts corresponding to the *Xio1* gene accumulated preferentially at 9 h after treatment with RaxX-sY but not RaxX. Taken together, our results suggest that *Xio1* is a novel *Oryza*-specific orphan gene having unknown function during the XA21-mediated immune response.

### Os09g13440.1 Is a Major Transcript of *Xio1*

In *O. sativa* ssp. *japonica* and *O. glumipatula*, possible alternative splicing forms were predicted and annotated in the databases of the Rice Genome Annotation Project^3^ and Ensemble Plants (see text footnote 1) (Supplementary Table 1). Five exons from *Xio1* are spliced together, resulting in Os09g13440.1, which encodes the putative full-length Xio1 protein of 24.9 kDa (Supplementary Figure 2A). Based on a database prediction, the Os09g13440.2 transcript results from the alternative splicing of a 95-bp alternative exon within intron II of the *Xio1* gene. The inclusion of the alternative exon in Os09g13440.2 provides the translation termination at the end of the exon, leading to a shorter Os09g13440.2 transcript encoding a protein of 21.8 kDa. To investigate whether both predicted alternatively spliced transcripts, Os09g13440.1 and Os09g13440.2, are expressed in *japonica* variety Kitaake, we performed RT-PCR analysis after treatment of RaxX or RaxX-sY in XA21/Kit plants (Supplementary Figure 2B). Primer sets specific to Os09g13440.1 and Os09g13440.2 were used (Supplementary Figure 2A). PR10 was significantly induced in RaxX-sY-treated XA21/Kit plants, indicating that RaxX-sY peptide treatment successfully induced the XA21-mediated immune response. Os09g13440.1-specific product of 490 bp was preferentially amplified from RaxX-sY-treated XA21 plants. However, although we tried several times, our attempt to amplify Os09g13440.2 failed. These results suggest that Os09g13440.1 is a major transcript of *Xio1* in *O. sativa* ssp. *japonica*.

### *Xio1* Is Intrinsically Disordered Protein Localized in the Nucleus and the Cytosol

**FIGURE 3:**
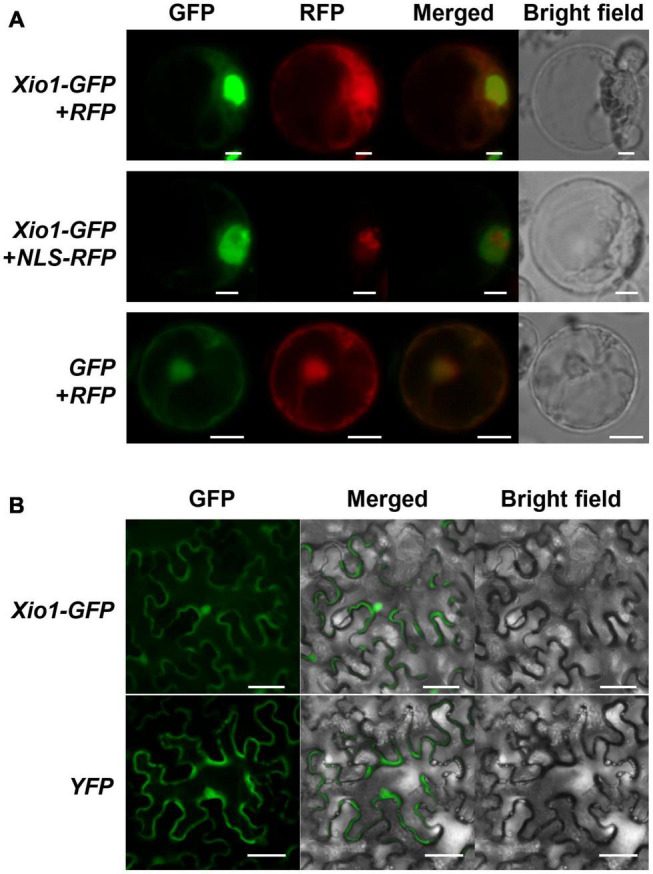
Subcellular localization of C-terminal GFP tagged Xio1 (Xio1-GFP) in rice and tobacco. **(A)** Microscopic analysis of Xio1-GFP in rice protoplasts. Xio1-GFP and RFP (upper panel), Xio1-GFP and NLS-RFP (middle panel), and GFP and RFP (lower panel) were transiently expressed in rice protoplasts using PEG-mediated transformation. Protoplasts were incubated at 25°C for 1 day after transformation and fluorescing cells were visualized using a Nikon fluorescent microscope with a C-FL-C FITC (excitation 465 to 495 nm) for GFP and a C-FL-C TRITC (excitation 537 to 552 nm) for RFP. The merged yellow signal indicates colocalization of GFP and RFP. Scale bar indicates 10 μm. **(B)** Microscopic analysis of Xio1-GFP within the leaf epidermal cells of tobacco. Xio1-GFP and yellow fluorescent protein (YFP) were transiently expressed within the leaf epidermal cells of tobacco using Agrobacterium-mediated infiltration. Tobacco leaves were maintained 1 day after transformation and fluorescing cells were visualized using a Nikon fluorescent microscope with a C-FL-C FITC (excitation 465 to 495 nm) for GFP and YFP. Scale bar indicates 50 μm. All experiments were repeated at least three times and shown with representative images.

Xio1 is a basic protein with pI 10.08 of 230 amino acids not carrying a predicted N-terminal signal peptide. However, the prediction in IUPred3^4^ (Erdos et al., 2021) showed that C-terminal region of Xio1 with score above 0.5 could be regarded as disordered regions (red line) and disordered binding sites (blue line) (Supplementary Figure 3). In addition, it contains two putative conserved motifs, nuclear localization signals (NLS) and nuclear export signals (NES) predicted by the NLStradamus^5^ (Nguyen Ba et al., 2009) and NetNES 1.1^6^ (la Cour et al., 2004), respectively (Figure 1A). The putative motifs suggest that Xio1 can translocalize into the nucleus for its novel function. To determine its subcellular location experimentally, Xio1 was tagged with green fluorescent protein (GFP) at its C-terminus and transiently expressed in rice protoplasts using PEG-mediated transformation (Figure 3A). In rice protoplasts co-transformed with *Xio1-GFP* and control *RFP* constructs, all transformed cells displayed identical subcellular localization patterns, characterized by GFP fluorescence mainly located in the nucleus-like compartment but also in the cytosol at a lower level (Figure 3A, *Xio1-GFP* + *RFP*). When *NLS-RFP* construct encoding a nuclear localization signal (NLS)-tagged RFP was co-delivered as a nucleus marker, Xio1-GFP was co-localized with NLS-RFP, indicating the nuclear localization of the Xio1 protein (Figure 3A, *Xio1-GFP* + *NLS-RFP*). To confirm its location, *Xio1-GFP* was transiently expressed in tobacco leaf epidermal cells using Agrobacterium-mediated infiltration (Figure 3B). In infiltrated leaves, fluorescence microscopy detected Xio1-GFP in the nucleus and the cytosol, which is consistent with its localization observed in rice protoplasts.

### Expression Analysis of *Xio1*

**FIGURE 4:**
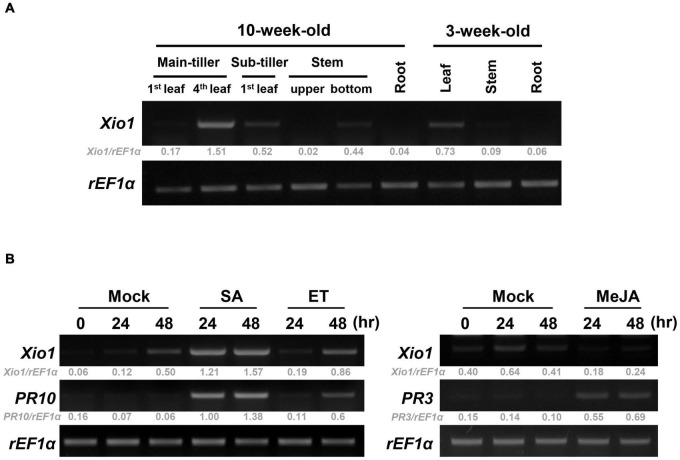
Expression analysis of *Xio1* in rice plants. **(A)** Developmental and organ-specific expression of *Xio1* gene. Various tissues were harvested from 10-week- and 3-week-old Kitaake plants. RT-PCR analysis was performed with *Xio1*-specific primers using total RNA extracted from various tissues. *rEF1*α was used as an internal control. **(B)** Expression pattern of *Xio1* in response to exogenous applications of phytohormones, salicylic acid (SA) and ethephon (ET) (left panel), and methyl jasmonic acid (MeJA) (right panel). For the phytohormone treatments, leaf sections (1.5–2.0 cm) were prepared from Kitaake plants and equilibrated overnight in 6-well cell culture plates under constitutive light. Then, leaf sections were treated with 200 μM SA, 1 mM ET, or 100 μM MeJA for indicated time points. *PR10* and *PR3* were used as a positive control for SA/ET and MeJA treatment, respectively. *rEF1*α was used as an internal control. Experiments were repeated three times, with similar results. Relative gray value of RT-PCR bands was quantified by ImageJ.

To investigate the unknown function of Xio1, we analyzed the expression pattern of *Xio1* in various tissues of 10- and 3-week-old Kitaake plants using RT-PCR (Figure 4A and Supplementary Figure 4). The transcripts of *Xio1* were detected at relatively high levels in fully expanded leaves (4th leaf) from main-tiller, but at significantly low levels in stem. The expression of *Xio1* in leaf tissue was also observed in 3-week-old plants. *Xio1* was barely expressed in both roots of 10-week-old and 3-week-old rice. Overall, *Xio1* displayed relatively high expression in mature leaf tissue.

The expression pattern of *Xio1* was also examined after treatments with phytohormones of salicylic acid (SA), ethylene-releaser ethephon (ET), and methyl jasmonic acid (MeJA/JA), which play key roles in coordinating plant immune responses (Figure 4B). *PR10* and *PR3* were used as marker genes for SA/ET- and MeJA-dependent signaling pathways, respectively (Xu et al., 1996; Jwa et al., 2001; Sathe et al., 2019), and demonstrated that phytohormone treatments were successful in the detached leaf bioassay. *Xio1* expression was induced to varying degrees by SA or ET (Figure 4B, left panel). Particularly, strong and rapid induction was observed by SA treatment. However, after MeJA treatment, *Xio1* expression was repressed to barely detectable levels repeatedly (Figure 4B, right panel), suggesting that there are antagonistic impacts of SA/ET and MeJA in its expression.

### Overexpression of *Xio1* Resulted in Growth Retardation

**FIGURE 5:**
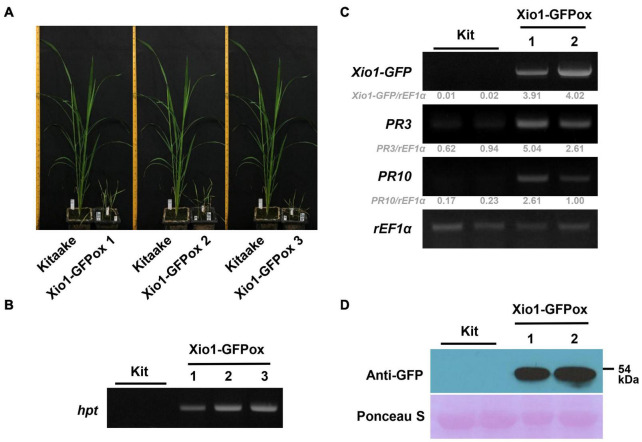
Generation of transgenic rice plants overexpressing Xio1-GFP. **(A)** Growth comparison between Kitaake and three independent transgenic lines (Xio1-GFPox T_0_, line 1, 2, and 3) overexpressing *Xio1* under normal greenhouse conditions. Photographs were taken 6 weeks after transferring to soil. **(B)** Genotyping of transgenic Xio1-GFPox (T_0_, line 1, 2, and 3) was performed using the *hpt*-specific primers, HygB_For and HygB_Rev. Genomic DNAs were extracted from Kitaake and Xio1-GFPox (T_0_, line 1, 2, and 3). **(C)** Overexpression of *Xio1-GFP* in the transgenic Xio1-GFPox (T_0_, line 1 and 2). RT-PCR analysis were performed using specific primers for *Xio1-GFP*, *PR3*, and P*R10*. *rEF1*α was used as an internal control. Relative gray value of RT-PCR bands was quantified by ImageJ. **(D)** Accumulation of Xio1-GFP in the transgenic Xio1-GFPox (T_0_, line 1 and 2). Western blot analysis was performed with anti-GFP antibody after total proteins were extracted from Kitaake and Xio1-GFPox. Rubisco large subunit stained with Ponceau S served as a loading control.

In order to examine the molecular function of Xio1 *in planta*, Agrobacterium-mediated transformation was performed with the overexpression construct, Ubi:Xio1-GFP/pC1300. Numbers of putative transgenic Kitaake plants (Xio1-GFPox) were generated, and they were phenotypically normal in root-inducing MS medium. However, all putative transgenic plants displayed growth retardation and eventually died approximately 2 months after transfer to potting soils in greenhouse. Despite many attempts, we were able to neither maintain the transgenic plants nor harvest seeds from any of them. Alternatively, we decided to analyze the putative T_0_ transgenic lines (Figure 5). Three putative independent transgenic Kitaake (Xio1-GFPox T_0_, line 1, 2, and 3) were generated and moved to soils in the greenhouse. After 6 weeks, all of them displayed growth retardation compared to the wild type Kitaake plant (Figure 5A). The putative transgenic plants were subjected to PCR-based selection using the *hpt* gene-specific primer set, HygB_For and HygB_Rev. All of them generated a *hpt*-specific 555 bp amplicon (Figure 5B), indicating that transgenic lines carrying the overexpression construct were generated.

We next examined the transcript level of *Xio1-GFP* in the transgenic plants (Figure 5C). In RT-PCR analysis, *Xio1-GFP*-specific primers (Figure 5C, *Xio1-GFP*) for transgene *Xio1-GFP* were used to determine its overexpression. As expected, significant amounts of *Xio1-GFP* transcripts were detected in the transgenic lines (T_0_), Xio1-GFPox 1 and 2, indicating that the *Xio1-GFP* transgene was constitutively overexpressed. In addition, we also investigated the expression levels of two defense-related marker genes, *PR3* and *PR10* (Xu et al., 1996; Jwa et al., 2001; McGee et al., 2001; Sathe et al., 2019), in the Xio1-GFPox lines (Figure 5C). Both *PR3* and *PR10* genes were significantly induced in the transgenic lines compared with the wild type Kitaake, suggesting that Xio1-GFP overexpression triggered defense response, regardless of *Xoo* inoculation. The transgenic lines (Xio1-GFPox T_0_, line 1 and 2) were assayed for the accumulation of the fusion protein, Xio1-GFP, by Western blot analysis using the anti-GFP antibody (Figure 5D). In the two lines, a significant accumulation in Xio1-GFP was observed, compared to the wild type Kitaake. These results indicated that transgenic lines overexpressing *Xio1-GFP* were successfully generated and that the phenotype of growth retardation and induction of defense-related marker genes should be caused by Xio1-GFP accumulation.

### Overexpression of *Xio1* Displayed Enhanced Resistance to *Xoo* and Reactive Oxygen Species Accumulation

**FIGURE 6:**
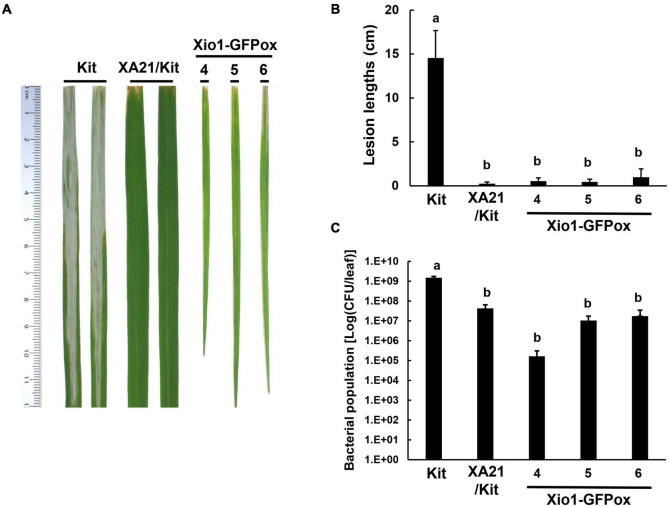
Transgenic rice plants overexpressing Xio1-GFP (Xio1-GFPox) exhibited enhanced resistance to *Xoo.* **(A)** Picture of representative rice leaves taken at 12 days after *Xoo* inoculation. From left to right: Kitaake (Kit), transgenic Kitaake overexpressing XA21 (XA21/Kit), transgenic Kitaake overexpressing Xio1-GFP (Xio1-GFPox). **(B)** Lesion lengths were measured in Kitaake, XA21/Kit, and transgenic Xio1-GFPox 12 days after *Xoo* inoculation. **(C)** *Xoo* populations were determined at 12 days after inoculation in Kitaake, XA21/Kit, and transgenic Xio1-GFPox. The error bars represent standard deviation values obtained from the three samples. Different letters indicate significant differences at *p* < 0.05.

Another three independent Xio1-GFPox lines (T_0_, line 4, 5 and 6) were generated and all of them generated a *Xio1-GFP*-specific 792 bp amplicon (Supplementary Figure 5A). The transgenic lines displayed significantly increased *Xio1* transcript and protein levels (Supplementary Figures 5B,C). Then, 6-week after transferring to soil in our greenhouse, the three Xio1-GFPox lines were inoculated with *Xoo* (Figure 6). They exhibited significantly enhanced resistance to *Xoo* with very short lesions of approximately 0.47–0.96 cm compared to Kitaake with lesion lengths of approximately 11.10–20.10 cm (Figures 6A,B). The bacterial population also correlated well with lesion length measurements (Figure 6C). *Xoo* populations in transgenic lines reached approximately 1.66 × 10^5^ – 1.75 × 10^7^ colony-forming units per leaf (cfu/leaf), whereas the population in Kitaake (Kit) plants reached to more than 1.29 × 10^9^ cfu/leaf. The inoculation experiment was repeated using different batch of transgenic lines, 7 and 8, with similar results (Supplementary Figure 6). These results demonstrated that *Xio1* overexpression conferred enhanced resistance to *Xoo* under our greenhouse condition.

**FIGURE 7:**
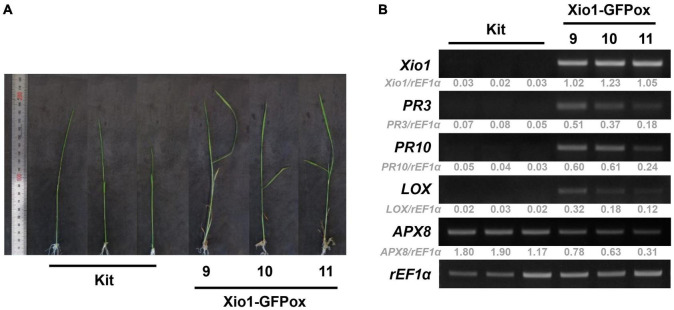
Expression analysis of defense-related genes in transgenic Xio1-GFPox grown on MS media. Photograph **(A)** and expression analysis **(B)** of defense-related genes of approximately 2-week-old Kitaake (Kit) and Xio1-GFPox lines (9, 10, and 11) grown on MS media in sterilized glass container under growth chamber condition. RT-PCR analysis was performed using specific primers for *Xio1*, *PR3*, *PR10*, lipoxygenase (*LOX*), and ascorbate peroxidase 8 (*APX8*) genes. *rEF1*α gene was used as an internal control. Relative gray value of RT-PCR bands was quantified by ImageJ.

To minimize the possibility that unknown environmental stresses in our greenhouse, instead of *Xio1* overexpression, induced stress-related responses in Xio1-GFPox lines, the next batch of five independent Xio1-GFPox lines (T_0_, line 9 to 13) were investigated in an environmentally controlled growth chamber. The transgenics and Kitaake control were grown in sterilized glass container containing MS medium and maintained under growth chamber condition. Two weeks (line 9, 10, and 11) and 3 weeks (line 12 and 13) after transferring to MS medium, the expression patterns of defense-related genes were examined (Figure 7 and Supplementary Figure 7). In both stages, two *PR* genes, *PR3* and *PR10*, were significantly induced in the transgenic lines. Interestingly, the lipoxygenase (*LOX*) gene encoding a lipid peroxidation-related enzyme also displayed high level expression in all Xio1-GFPox lines. In contrast, ascorbate peroxidase 8 (*APX8*) encoding an antioxidative enzyme was reduced. These results suggest that *Xio1* overexpression elicited defense responses to *Xoo* by expressing various defense- and antioxidant-related genes directly or indirectly.

**FIGURE 8:**
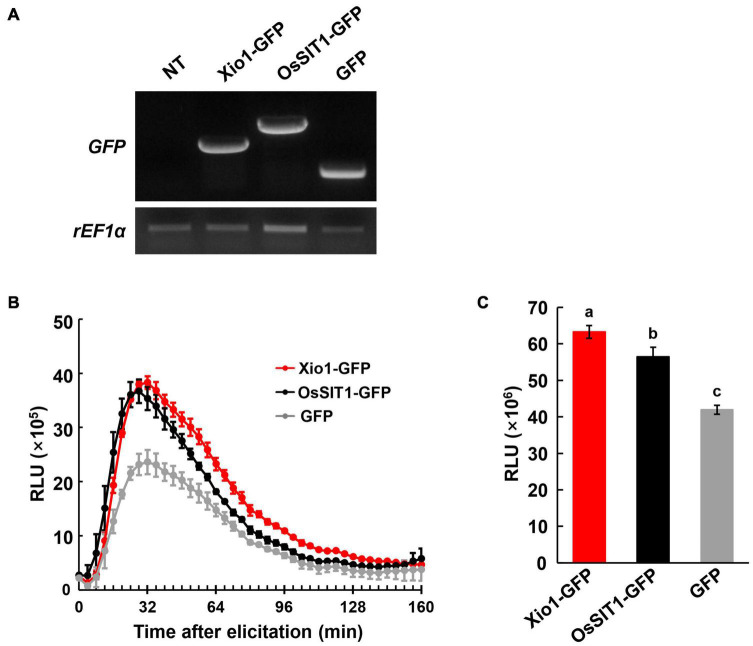
Elevated ROS levels observed in protoplasts transiently overexpressing *Xio1-GFP*. **(A)** Expression of *Xio1-GFP*, *OsSIT1-GFP*, and *GFP* in rice protoplasts. Protoplasts were transformed with each construct (*Xio1-GFP*, *OsSIT1-GFP*, and *GFP*). Non-transformed protoplasts (NT) were included as a control. RT-PCR analysis was performed using specific primers for *Xio1-GFP*, *OsSIT1-GFP*, *GFP*, and *rEF1*α. **(B)** Dynamics of ROS burst in rice protoplasts expressing *Xio1-GFP*, *OsSIT1-GFP*, and *GFP*. ROS was measured in a luminol-based chemiluminescence assay and represented as accumulated relative luminescence units (RLU). The measurement was performed at the indicated time points after luminol treatments. **(C)** Accumulated relative luminescence units (RLU) from 0 to 152 min after treatment. Different letters indicate significant differences at *p* < 0.01. These experiments were performed more than four times, and a representative result is shown here.

Because of the premature death of all Xio1-GFPox lines, it was impossible to further analyze the transgenic plants for investigating antioxidant enzymes, in conjunction with ROS accumulation. ROS homeostasis has been successfully analyzed in various protoplast systems including Arabidopsis, *Cucumis sativus* (cucumber), and *Solanum lycopersicum* (tomato) (Ondrej et al., 2010; Tiew et al., 2015; Gandhi et al., 2020). Therefore, to measure extracellular H_2_O_2_ production, we performed the luminol-based chemiluminescence assay in rice protoplasts. Rice salt intolerance 1 (*OsSIT1*) was used as a positive control in the assay because its overexpression prompted the accumulation of ROS in rice (Li et al., 2014). Xio1-GFP, OsSIT1-GFP, and GFP were successfully expressed in rice protoplasts transformed with each construct (Figure 8A). In Figures 8B,C, protoplasts expressing GFP control displayed slightly elevated H_2_O_2_, which is commonly observed in freshly isolated protoplasts (Tiew et al., 2015). In contrast, dramatically increased H_2_O_2_ was observed after transformation with *OsSIT1-GFP*, indicating that the luminol-based chemiluminescence assay in protoplasts worked successfully. Xio1-GFP expression was also significantly increased H_2_O_2_ production at a higher level than that caused by OsSIT1-GFP.

### Intergeneric Transfer of *Xio1* to *Arabidopsis* Dose Not Confer Resistance to *Pst* DC3000

**FIGURE 9:**
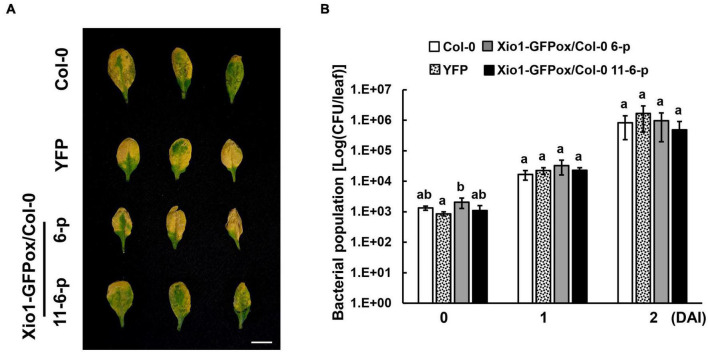
Disease symptoms and bacterial populations in Arabidopsis overexpressing *Xio1-GFP* (Xio1-GFPox/Col-0) after infiltration with *Pst* DC3000. **(A)** Symptoms of 4-week-old wild-type Arabidopsis (Col-0), transgenic Col-0 lines overexpressing YFP (YFP), and transgenic Col-0 lines overexpressing Xio1-GFP (Xio1-GFPox/Col-0, progenies of lines 6 and 11-6) 3 days after syringe infiltration with *Pst* DC3000. Representative rosette leaves were photographed. Scale bar, 1 cm. **(B)** Growth of *Pst* DC3000 in Arabidopsis Col-0, YFP, and Xio1-GFPox/Col-0 (progenies of lines 6 and 11-6). Bacterial titers were evaluated at 0, 1, and 2 days after inoculation (DAI). The error bars represent standard deviation values obtained from the three samples. Different letters indicate significant differences at *p* < 0.05. Experiments were repeated three times, with similar results.

To investigate whether *Xio1* plays distinct role in disease resistance in non-Oryza plant species, *Xio1* was overexpressed in Arabidopsis Col-0. We generated 12 independently transformed transgenic Arabidopsis lines (Xio1-GFPox/Col-0, T_1_) carrying 35S:Xio1-GFP/pEG103 construct (Supplementary Figure 8A). All of them displayed significant expression of *Xio1-GFP* (Supplementary Figure 8B). Of these, two lines (6 and 11) with relatively higher expression of *Xio1-GFP* were selected for further analysis. Western blot analysis performed with their progenies (6-1 and 11-13-1) exhibited high accumulation of fusion protein, Xio1-GFP (Supplementary Figure 8C). In the transgenic Arabidopsis, fluorescence from Xio1-GFP was detected in the nucleus and the cytosol (Supplementary Figure 9), consistent with previous observations in rice protoplasts and tobacco leaves (Figure 3). However, unlike in rice, *Xio1-GFP* overexpression in Arabidopsis did not cause any obvious morphological changes, including growth retardation (Supplementary Figures 10A,B) and increased expression of *PR* genes, *AtPR1* and *AtPR4* (Supplementary Figure 10C), when compared to non-transgenic Col-0 plants. In addition, after challenging with a virulent strain of *Pseudomonas syringae* pv. *tomato* DC3000 (*Pst* DC3000), they did not display any significant difference in disease symptom and bacterial population compared with non-transgenic Col-0 (Figure 9). This indicated that *Oryza*-specific orphan gene, *Xio1*, was not able to induce disease resistance in non-*Oryza* plant species, Arabidopsis.

## Discussion

Only a few lineage-specific orphan genes have been characterized in plants because of difficulties in predicting their functional role using comparative genomics. In this study, we characterized an *Oryza*-specific orphan gene, *Xio1*, involved in resistance to *Xoo* in rice.

After the genus *Oryza* had several genomes sequenced (Stein et al., 2018), it is now considered as a model system for identification and evolution study of lineage-specific orphan genes. *Xio1* homologous sequences were found only in the AA genome type, including *O. sativa* ssp., *japonica*, *O. glumipatula*, *O. rufipogon*, *O. barthii*, *O. meridionalis*, and *O. sativa* ssp. *indica*, but not in *O. punctata* (BB genome type) and *O. brachyantha* (FF genome type), or any other genome available in a public database. Because the BB genome is evolutionally the closest to the AA genome (Stein et al., 2018), functional *Xio1* origination can be timed to less than approximately 6.7 million years ago, which suggests that *Xio1* could be young genes under strong selective pressure for rapid evolution. Interestingly, *XA21* was derived from a wild African species *O. longistaminata* (Song et al., 1995), not carrying *Xio1* homologous sequences. Therefore, we assume that *Xio1* is induced during defense responses to pathogens, not specific to XA21/*Xoo* (raxX-sY) interaction. There are reports showing that a few orphans in plants are the result of rapid co-evolution with their pathogens. In wheat, Septoria-responsive taxonomically restricted gene 6 (TaSRTRG6) and TaSRTRG7 interacted with potential effector candidates secreted by *Zymoseptoria tritici* (Brennan et al., 2020). Orphan secreted protein 24 (Osp24) in *F. graminearum* targets TaSnRK1α for degradation, and TaFROG competes with Osp24 to bind with TaSnRK1α and prevents its degradation (Jiang et al., 2020). The bacterial blight resistance gene Xa7 in rice is a recently cloned orphan gene harboring promoter trap for AvrXa7 secreted by *Xoo* (Wang et al., 2021). These results suggest that active adoption and molecular evolution of orphan genes might be common events during interaction between plants and their pathogens.

**FIGURE 10:**
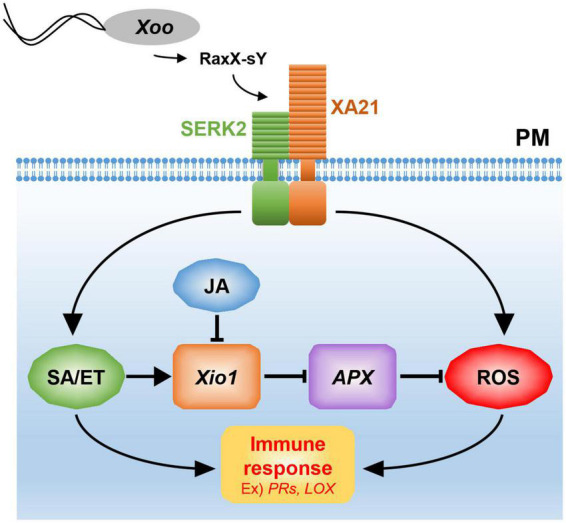
A model for Xio1 in XA21-mediated immunity. XA21 constitutively associates with somatic embryogenesis receptor kinase 2 (SERK2) (Chen et al., 2014). A ROS-scavenging enzyme, APX, catalyzes the removal of H_2_O_2_ (ROS) (Durner and Klessig, 1995; Mittler et al., 1998). XA21-mediated immunity is triggered by recognition of the sulfated microbial peptide, RaxX-sY, secreted from *Xoo* (Pruitt et al., 2015). Upon the recognition, XA21 activates SA/ET-dependent defense responses and produces ROS rapidly (Yang et al., 2013; Pruitt et al., 2015). Activation of SA/ET-mediated signaling pathway induces a set of defense-related genes and *Xio1*, which negatively regulates *APX* expression. Down-regulation of *APX* leads to additional ROS accumulation, which in turn induces immune response including the expression of *PR* genes and *LOX*. SA/ET-JA antagonism is a key strategy for the expressional regulation of *Xio1*, although the molecular mechanism remains to be investigated.

*Xio1* expression was significantly induced by treatment of representative defense-related phytohormone SA. Resistance to *Xoo* was decreased in the transgenic XA21 plants expressing an SA hydroxylase degrading SA, suggesting that SA is required for XA21-mediated resistance to *Xoo* (Yang et al., 2013). RaxX21-sY activated the ethylene production in an XA21-dependent manner (Pruitt et al., 2015). Therefore, SA/ET-dependent defense responses activated by XA21 is likely responsible for the expression of *Xio1* (Figure 10). Interestingly, *Xio1* was reduced by the treatment of another defense-related phytohormone MeJA/JA. The antagonistic interaction between SA and JA plays a critical role in defense responses of many plant species including rice (Thaler et al., 2012). For example, SA treatment attenuated JA-induced expression of the rice PR10 gene (Takeuchi et al., 2011). Overexpression of rice NPR1 (non-expressor of pathogenesis-related genes 1) resulted in strong activation of SA-responsive genes and concomitant suppression of JA responsive genes (Yuan et al., 2007), and its silencing displayed elevated levels of JA and increased expression of JA biosynthetic genes (Li et al., 2013). Overall, our results suggest that the antagonistic interplay between SA/ET and JA is one of the key strategies for expressional regulation of *Xio1*.

Excessive production of ROS mediated by many phytohormones including SA and JA has been to known as one of the earliest induced defense responses in plants (Heller and Tudzynski, 2011). ROS performs a key immune function acting in defense, killing pathogens, and as signaling molecules to further activate immune responses (Qi et al., 2017). Therefore, in the absence of pathogens, a basal level of ROS should be maintained by the balance between ROS production and ROS scavenging. In the ROS scavenging system, APX catalyzes the removal of hydrogen peroxide (H_2_O_2_), one of the most abundant ROS subspecies, by converting it to H_2_O and O_2_ (Apel and Hirt, 2004; Heller and Tudzynski, 2011). Therefore, the expression and activity of APX are known to decline to maintain elevated H_2_O_2_ level during the defense response (Durner and Klessig, 1995; Mittler et al., 1998). Because *APX8* expression was reduced in all Xio1-GFPox lines, the H_2_O_2_ concentration in the transgenic lines might be maintained at a high level, presumably to allow the activation of the defense signaling pathway including the expression of *PR* genes (Figure 10). Supporting this hypothesis, rice protoplasts overexpressing *Xio1* displayed significantly elevated H_2_O_2_ level. In addition, the APX decline and subsequent H_2_O_2_ elevation were reported to induce the expression of the *LOX* gene in Arabidopsis (Banuelos et al., 2008), which was also observed in all Xio1-GFPox lines. LOXs are involved in many biological processes including tuber development (Kolomiets et al., 2001), senescence (Seltmann et al., 2010), and disease resistance (Wang et al., 2008). Therefore, our results suggest that ROS accumulation caused by Xio1 expression in rice could be a result of uncoupling with ROS removal by cellular antioxidative mechanisms. Further study in the molecular mechanism will be elucidate how Xio1 is involved in the regulation of ROS signaling and defense-related genes.

Constitutive overexpression of *Xio1* in rice displayed significantly enhanced resistance but resulted in severe growth retardation and premature death. In most plants, strong defense activation usually comes at the expense of plant growth, which is commonly known as the growth-defense trade-off (Huot et al., 2014; Neuser et al., 2019). For example, overexpression of rice *WRKY67* and *MYC2* displayed enhanced resistance to *Magnaporthe grisea* and *Xoo*, leading to growth retardation (Uji et al., 2016; Vo et al., 2017). Although the exact mechanisms underlying growth-defense trade-offs need to be elucidated, many defense-related genes including *PR* genes were significantly induced in both transgenic plants overexpressing *WRKY67* or *MYC2*. Strong induction of defense-related genes, *PR3* and *PR10*, were also repeatedly observed in *Xio1*-overexpressing transgenic plants, suggesting that growth retardation might be a result of growth-defense trade-off caused by *Xio1* overexpression.

Contrary to the results in rice, overexpression of *Xio1-GFP* in non-*Oryza* plant species, Arabidopsis, did not display any growth retardation, increased expression of defense-related genes, and enhanced resistance to *Pst* DC3000. However, there are increasing reports that the functionality of at least some of orphans may be transferable to other species in which the particular orphan gene is not present. For example, Brassicaceae-specific orphan genes, *EWR1* (for enhancer of vascular wilt resistance) from Arabidopsis and *Brassica oleracea*, confer enhanced resistance to wilt disease in tobacco, a member of the *Solanaceae* family (Yadeta et al., 2014). *Arabidopsis thaliana*-specific orphan Qua-Quine Starch (QQS) modulated carbon and nitrogen allocation in other species, *Glycine max* (soybean) (Li and Wurtele, 2015), and *Zea mays* (maize) and rice (Li et al., 2015). Overexpression of rice *Xa7* elicited cell death reaction not only in rice but also in tobacco (Wang et al., 2021). It can only be determined experimentally whether a certain orphan gene is transferable to other species. Considering that Xio1-GFP in Arabidopsis transgenic lines was successfully expressed, one possible explanation is that there could be a missing component such as an Arabidopsis structural/functional homolog of rice proteins interacting with Xio1.

Subcellular localization experiments performed with Xio1-GFP fusion in tobacco and Arabidopsis leaves, and rice protoplasts suggested that Xio1 plays its role in the nucleus. It cannot be concluded that the predicted NLS and NES motif in Xio1 are functional without further validating the motifs. However, some of orphan proteins are present in the nucleus, where they interact with transcription factors and regulate gene expression. *Arabidopsis thaliana*-specific orphan QQS forms a complex with the transcription factor nuclear factor Y subunit C4 (NF-YC4) and modulates transcription of target genes in the nucleus (Li et al., 2015). In wheat, the *Pooideae*-specific orphan protein TaFROG is another example of the orphan–transcription factor interaction. The intrinsically disordered nuclear protein interacts with SNF1-related kinase TaSnRK1α (Perochon et al., 2015). TaFROG also interacts with the NAC-like transcription factor TaNACL-D1, enhancing resistance to *F. graminearum* (Perochon et al., 2019). Therefore, it is possible that induction of defense-related genes observed in Xio1-GFPox lines could be a result of regulation through binding of transcription factors to a disordered binding site predicted at C-terminal of Xio1 in the nucleus.

In the current study, we present the functional characterization of *Oryza*-specific orphan gene, *Xio1*, specifically induced by *Xoo* in the XA21-dependent manner. Overexpression of *Xio1* displayed significantly enhanced resistance to *Xoo*, in company with constitutive expression of defense-related genes, suggesting that lineage-specific genes can be used as gene sources for crop improvements. Future studies will likely focus on identifying Xio1 interacting proteins to understand its putative function in the nucleus.

## Data Availability Statement

The datasets presented in this study can be found in online repositories. The names of the repository/repositories and accession number(s) can be found in the article/Supplementary Material.

## Author Contributions

HM and C-JP conceived and designed the experiments and wrote the manuscript. HM, A-RJ, and O-KK performed the experiments and analyzed the data. All authors read and approved the final manuscript.

## Conflict of Interest

The authors declare that the research was conducted in the absence of any commercial or financial relationships that could be construed as a potential conflict of interest.

## Publisher’s Note

All claims expressed in this article are solely those of the authors and do not necessarily represent those of their affiliated organizations, or those of the publisher, the editors and the reviewers. Any product that may be evaluated in this article, or claim that may be made by its manufacturer, is not guaranteed or endorsed by the publisher.

## Footnotes

1
https://plants.ensembl.org/

2
https://myhits.sib.swiss/cgi-bin/motif_scan

3
http://rice.uga.edu

4
https://iupred.elte.hu/

5
http://www.moseslab.csb.utoronto.ca/NLStradamus/

6
http://www.cbs.dtu.dk/services/NetNES/
