# Supplementary material for: Oryza-Specific Orphan Protein Triggers Enhanced Resistance to Xanthomonas oryzae pv. oryzae in Rice
Source: Front Plant Sci. 2022 Mar 10;13:859375. doi: 10.3389/fpls.2022.859375 (PMC8961030; doi:10.3389/fpls.2022.859375)
Supplement: Supplementary file 1 [file Data_Sheet_1.docx]

Supplementary Material

# Supplementary Figures


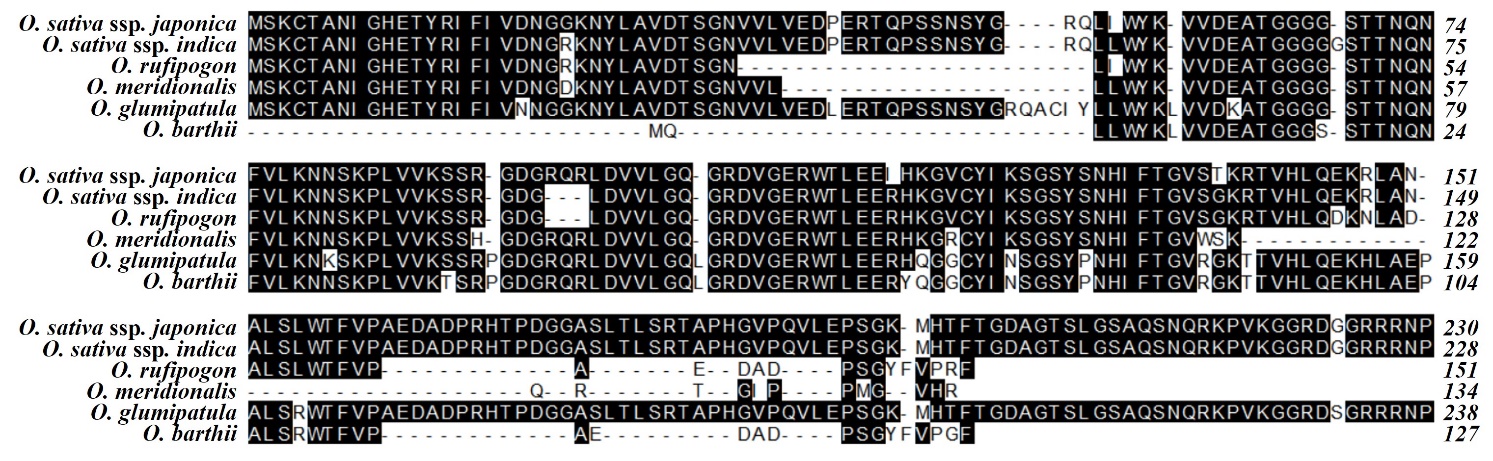


**Supplementary Figure 1.** Comparison of the predicted amino acid sequences of *Xio1* and its homologs from *Oryza* spp., *Oryza sativa* ssp. japonica, *O. sativa* ssp. indica, *O. rufipogon*, *O. meridionalis*, *O. glumipatula*, and *O. brathii*. DNASTAR MegAlign by ClustalW (weighted) method was used to generate the sequence alignment.


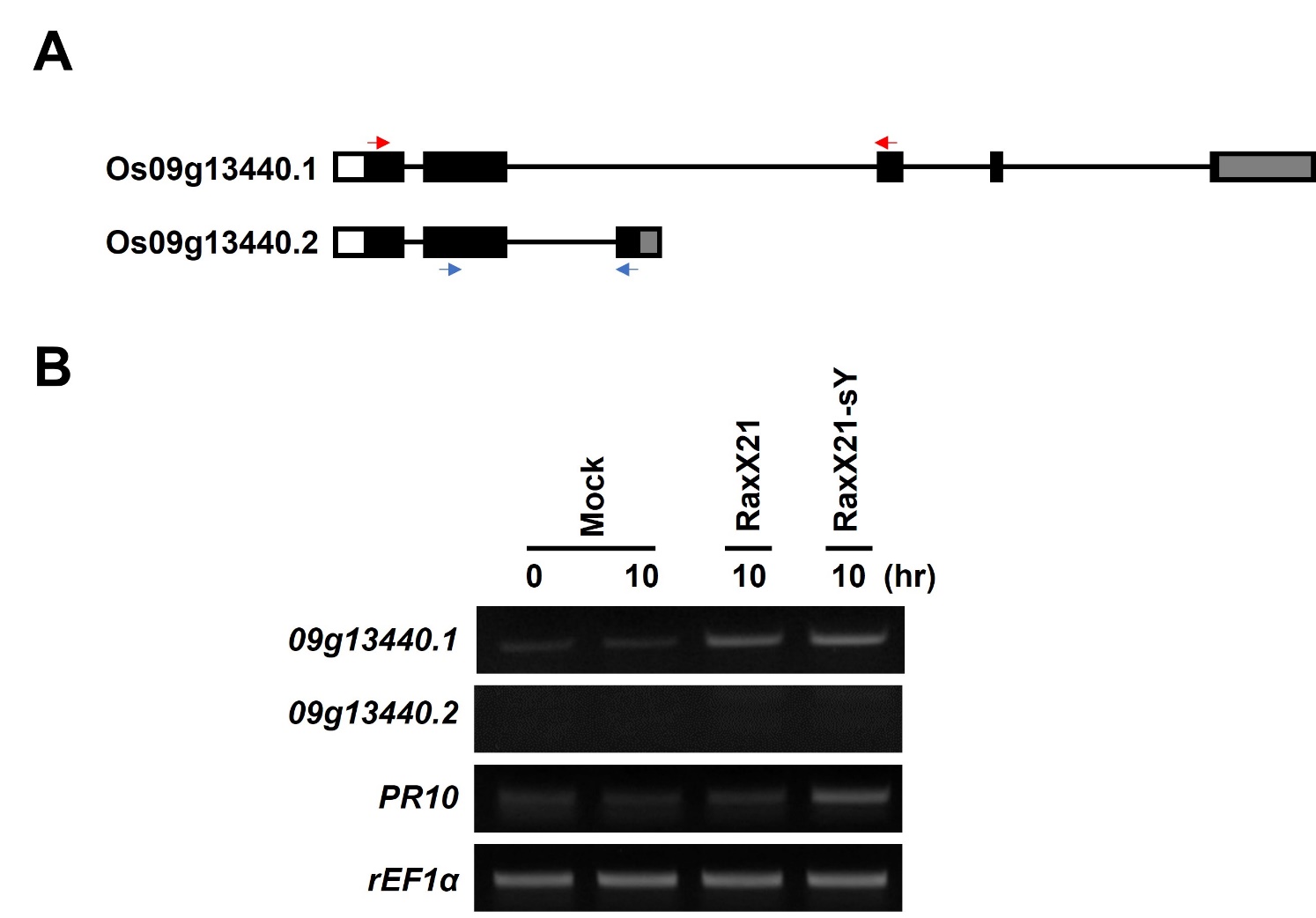


**Supplementary Figure 2.** *Xio1* is the only transcript among predicted alternative splicing forms. **(A)** The deduced gene structure of Os09g13440.1 (*Xio1*) and its possible alternative splicing form Os09g13440.2 predicted in Rice Genome Annotation Project (http://rice.uga.edu). Black box, exon; line, intron; white box, 5′ UTR; gray box, 3′ UTR. Specific primer sets to Os09g13440.1 (*Xio1*) and Os09g13440.2 were indicated with red and blue arrow, respectively. **(B)** Expression of Os09g13440.1 and Os09g13440.2 genes in XA21/Kit after treatments with a sulfated peptide RaxX (RaxX-sY) or inactive nonsulfated peptide (RaxX). RT-PCR analysis were performed using specific primer sets displayed in **(A)**. *rEF1α* and pathogenesis-related protein 10 (*PR10*) were used as an internal control and a positive control for RaxX-sY treatment, respectively. Experiments were repeated at least three times, with similar results.


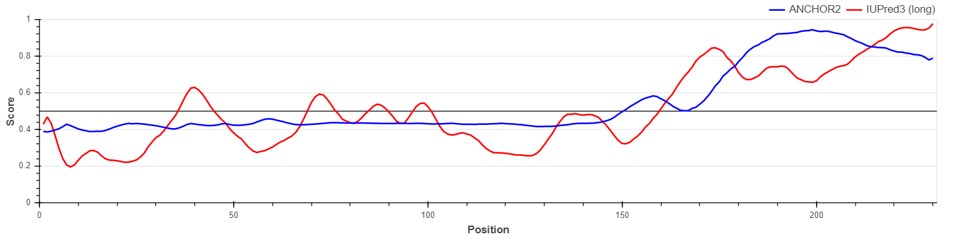


**Supplementary Figure 3.** Prediction of disordered region and disordered binding site in Xio1 using IUPred3 (https://iupred.elte.hu/) with default parameters.


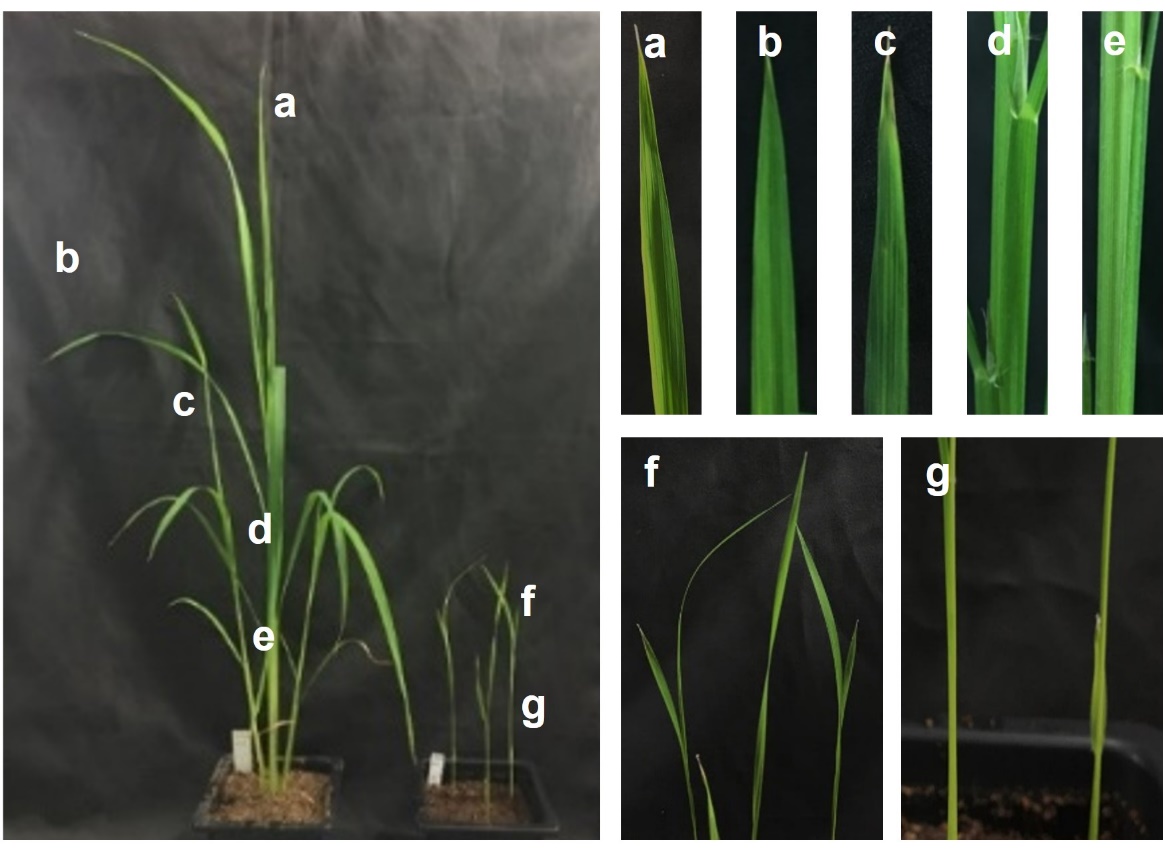


**Supplementary Figure 4.** Various tissues harvested from 10-week-old and 3-week-old rice plants. 10-week-old Kitaake plants: a, 1st leaf from main tiller; b, 4th leaf from main tiller; c, 1st leaf from sub-tiller; d, upper stem; e, lower stem. 3-week-old Kitaake plants: f, leaf; g, stem.

*
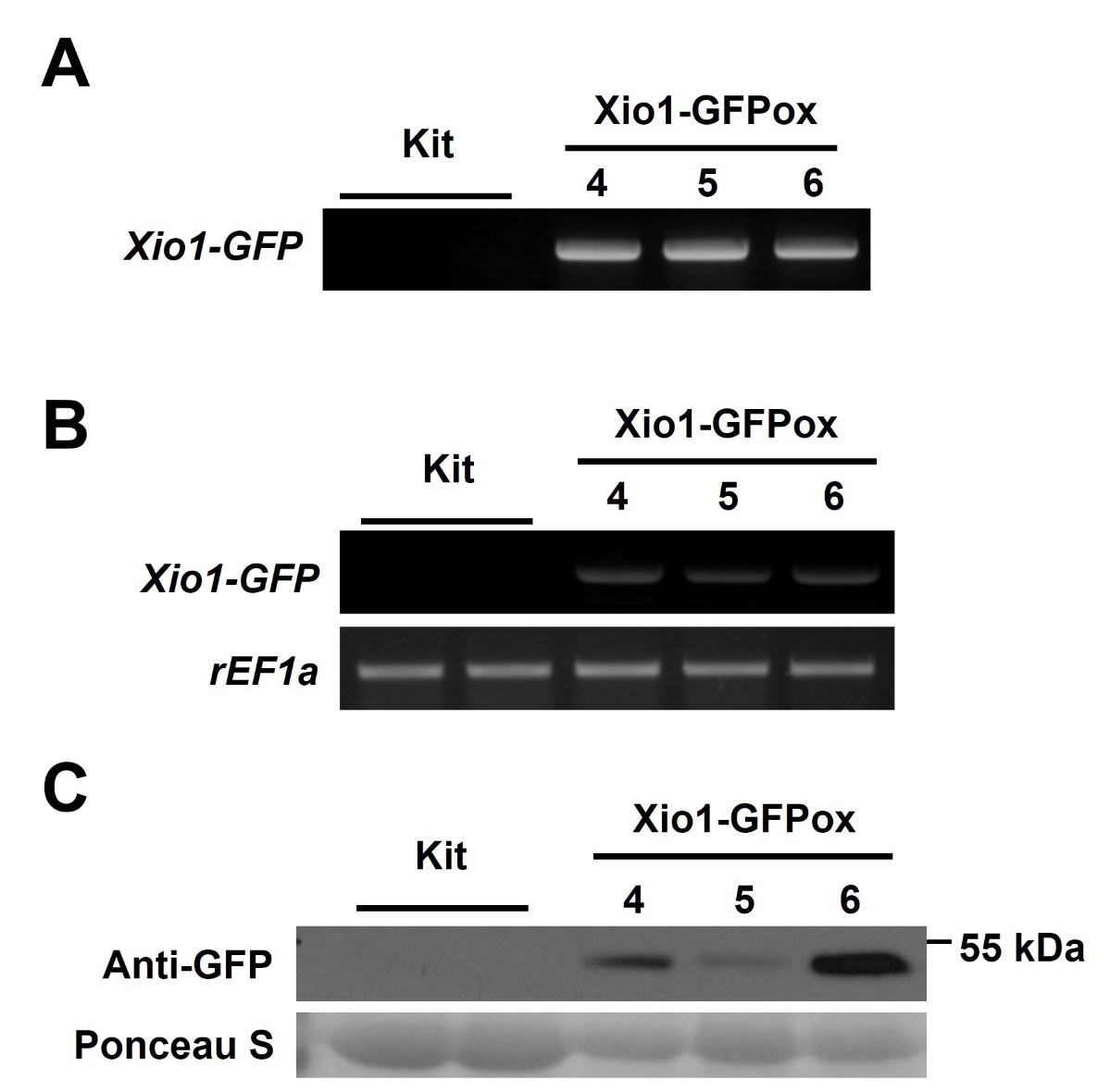
*

**Supplementary Figure 5.** Generation of transgenic rice plants overexpressing *Xio1-GFP*. **(A)** Genotyping of transgenic Xio1-GFPox (T_0_, line 4, 5, and 6). Genotyping was performed using the Xio1-GFP-specific primers from three independently transformed plants (Xio1-GFPox T_0_, line 4, 5, and 6). **(B)** Overexpression of *Xio1-GFP* in the transgenic Xio1-GFPox (T_0_, line 4, 5, and 6). RT-PCR analysis were performed using specific primers for *Xio1-GFP*. *rEF1α* was used as an internal control. **(C)** Accumulation of Xio1-GFP in the transgenic Xio1-GFPox (T_0_, line 4, 5, and 6). Western blot analysis was performed with anti-GFP antibody after total protein extracts were extracted from Kitaake and Xio1-GFPox. Rubisco large subunit stained with Ponceau S served as a loading control.


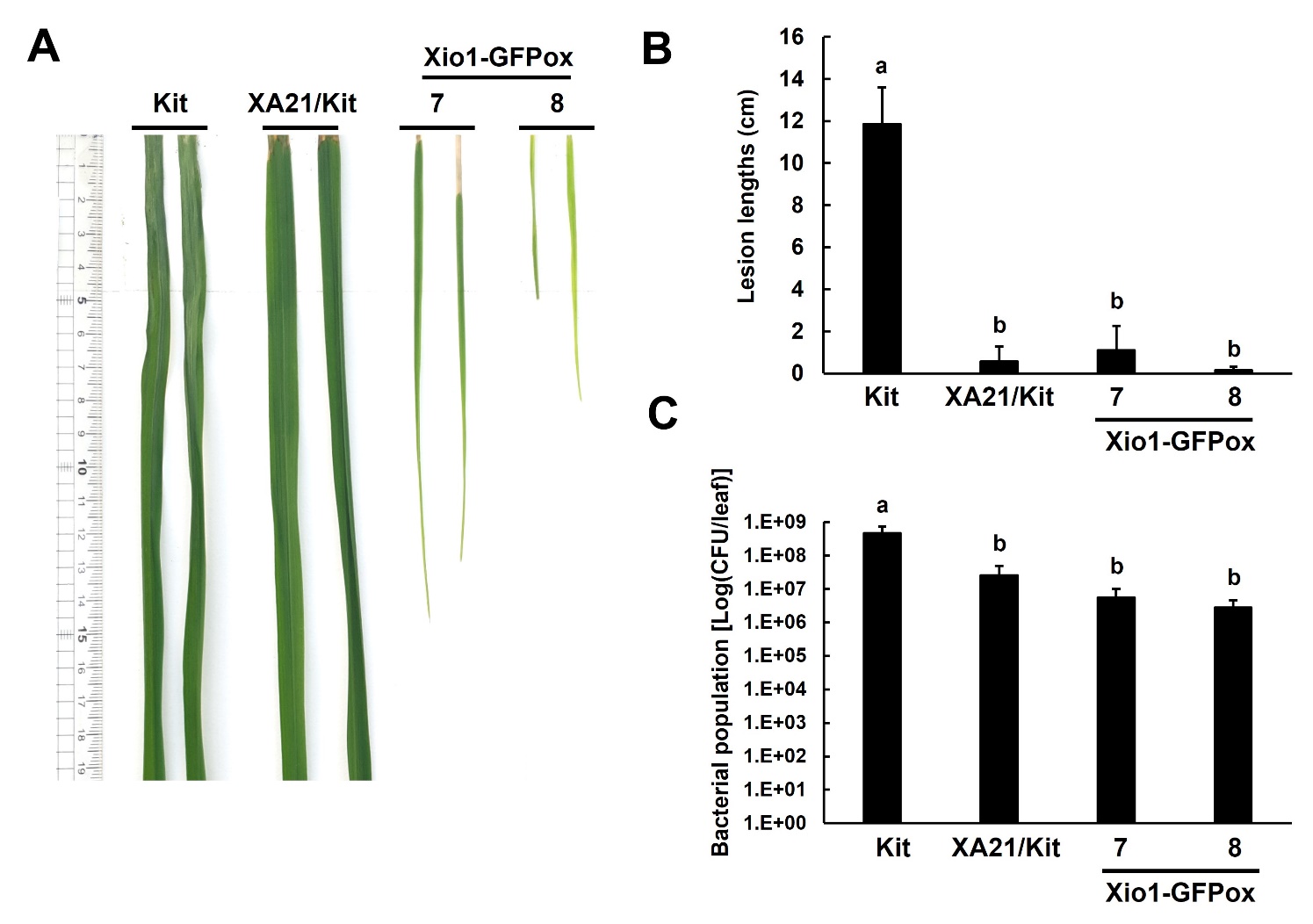


**Supplementary Figure 6.** Transgenic rice plants overexpressing *Xio1-GFP* (Xio1-GFPox) exhibit enhanced resistance to *Xoo.* **(A)** Picture of representative rice leaves taken at 12 days after *Xoo* inoculation. From left to right: Kitaake (Kit), transgenic Kitaake overexpressing XA21 (XA21/Kit), transgenic Kitaake overexpressing *Xio1-GFP* (Xio1-GFPox T_0_, line 7 and 8). **(B)** Lesion lengths were measured in Kitaake, XA21/Kit, and transgenic Xio1-GFPox (T_0_, line 7 and 8) 12 days after *Xoo* inoculation. **(C)** *Xoo* populations were determined at 12 days after inoculation in Kitaake, XA21/Kit, and transgenic Xio1-GFPox (T_0_, line 7 and 8). The error bars represent standard deviation values obtained from the three samples. Different letters indicate significant differences at p < 0.001.


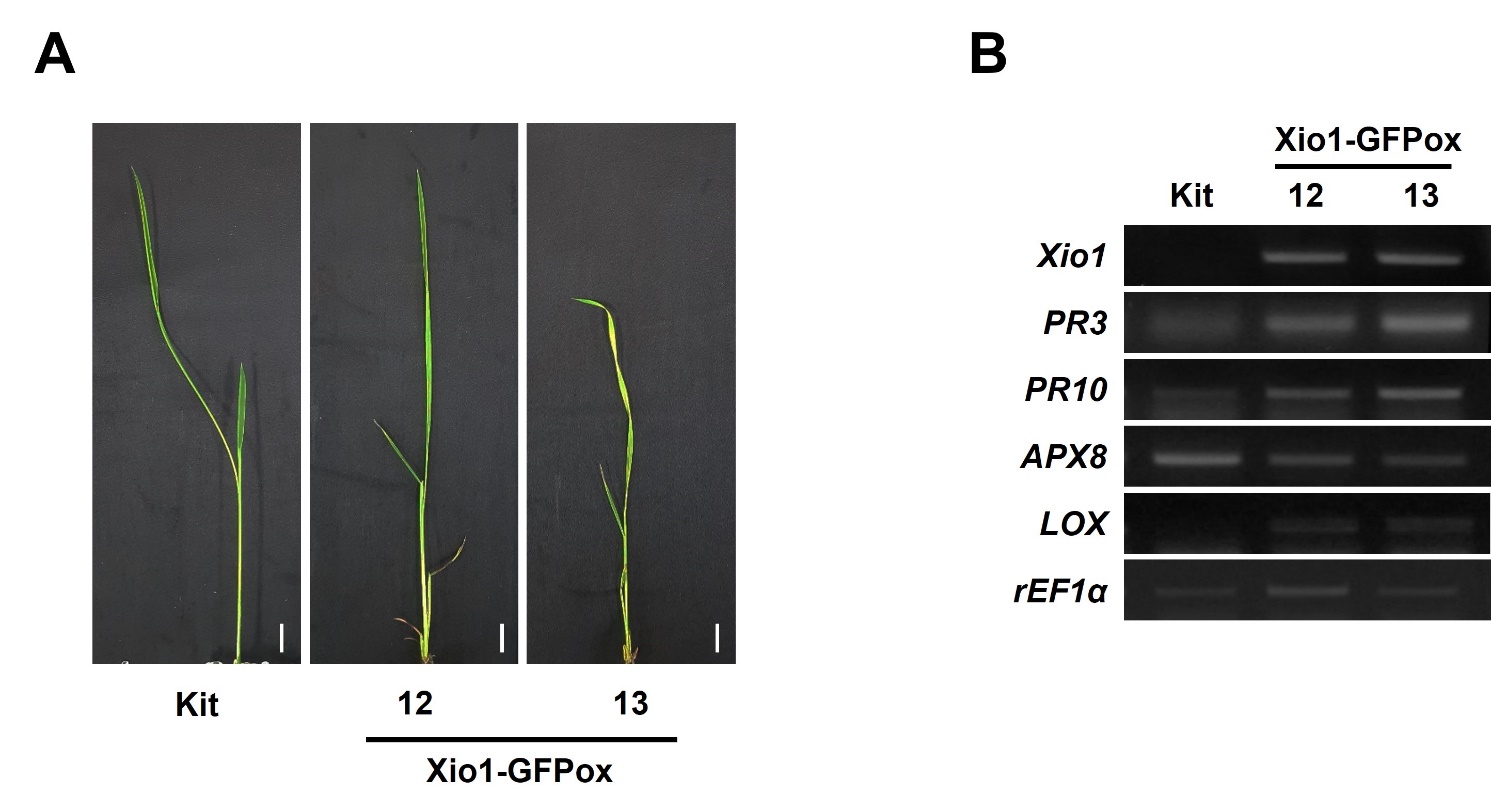


**Supplementary Figure 7.** Expression analysis of defense-related genes in transgenic Xio1-GFPox grown on MS media. Photograph **(A)** and expression analysis **(B)** of defense-related genes of approximately three-week-old Kitaake (Kit) and Xio1-GFPox (T_0_, line 12 and 13) grown on MS media in sterilized glass container under growth chamber condition. RT-PCR analysis was performed using specific primers for *Xio1*, *PR3*, *PR10*, lipoxygenase (*LOX*), and ascorbate peroxidase 8 (*APX8*) genes. *rEF1α* gene was used as an internal control. Scale bars, 1 cm.


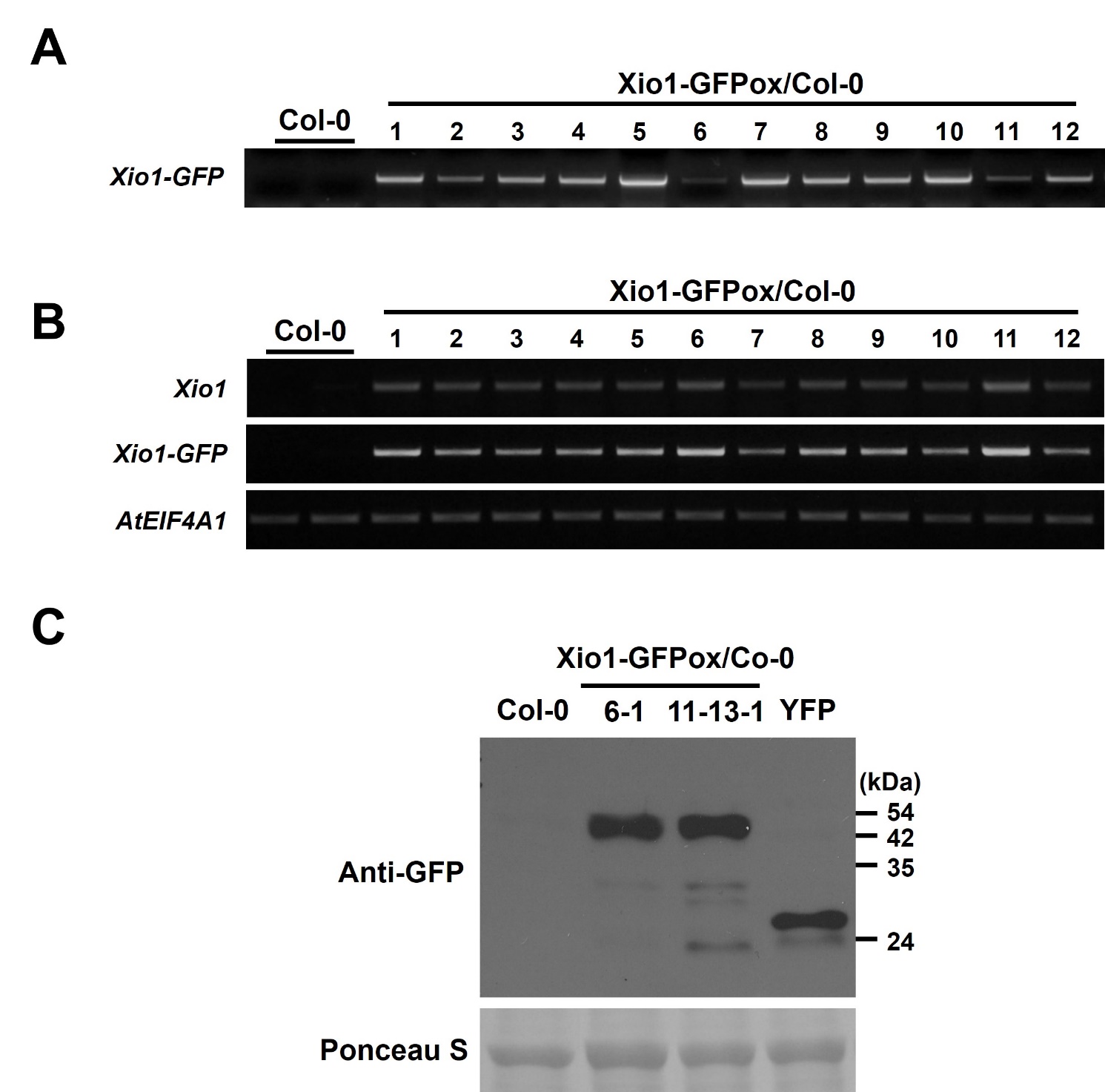


**Supplementary Figure 8.** Generation of transgenic Arabidopsis plants overexpressing *Xio1-GFP.* **(A)** Genotyping of transgenic Arabidopsis Col-0 overexpressing *Xio1-GFP* (Xio1-GFPox/Col-0). Genotyping was performed using the specific primers for *Xio1-GFP* from twelve independently transformed plants (Xio1-GFPox/Col-0 T_1_, lines 1 to 12). **(B)** Overexpression of *Xio1-GFP* in the transgenic Arabidopsis Xio1-GFPox/Col-0 lines. RT-PCR analysis were performed using specific primers for *Xio1* and *Xio1-GFP*. *AtEIF4A1* was used as an internal control. **(C)** Accumulation of Xio1-GFP in the transgenic Arabidopsis Xio1-GFPox/Col-0 lines. Western blot analysis was performed with anti-GFP antibody after total protein extracts were extracted from Col-0, Xio1-GFPox/Col-0, and YFP. Protein gel loading control was done by Ponceau S staining.


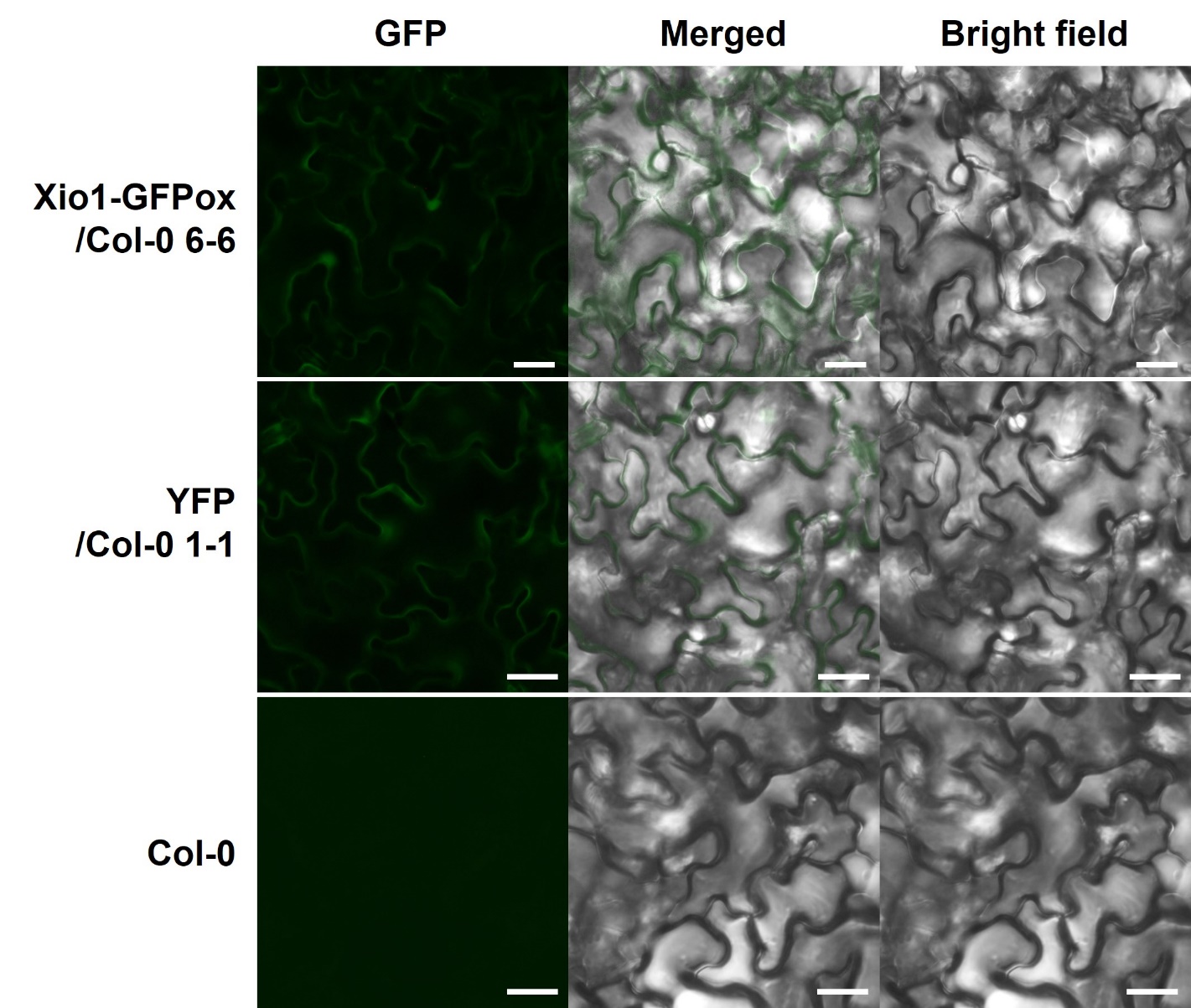


**Supplementary Figure 9.** Microscopic analysis of Xio1-GFP within the leaf epidermal cells of Arabidopsis. Photographs of Xio1-GFPox/Col-0, YFP/Col-0, and Col-0 were taken using a Nikon fluorescent microscope with a C-FL-C FITC (excitation 465 nm to 495 nm) for GFP and YFP. Scale bar indicates 50 μm. Experiments were repeated three times and shown with representative images.

**
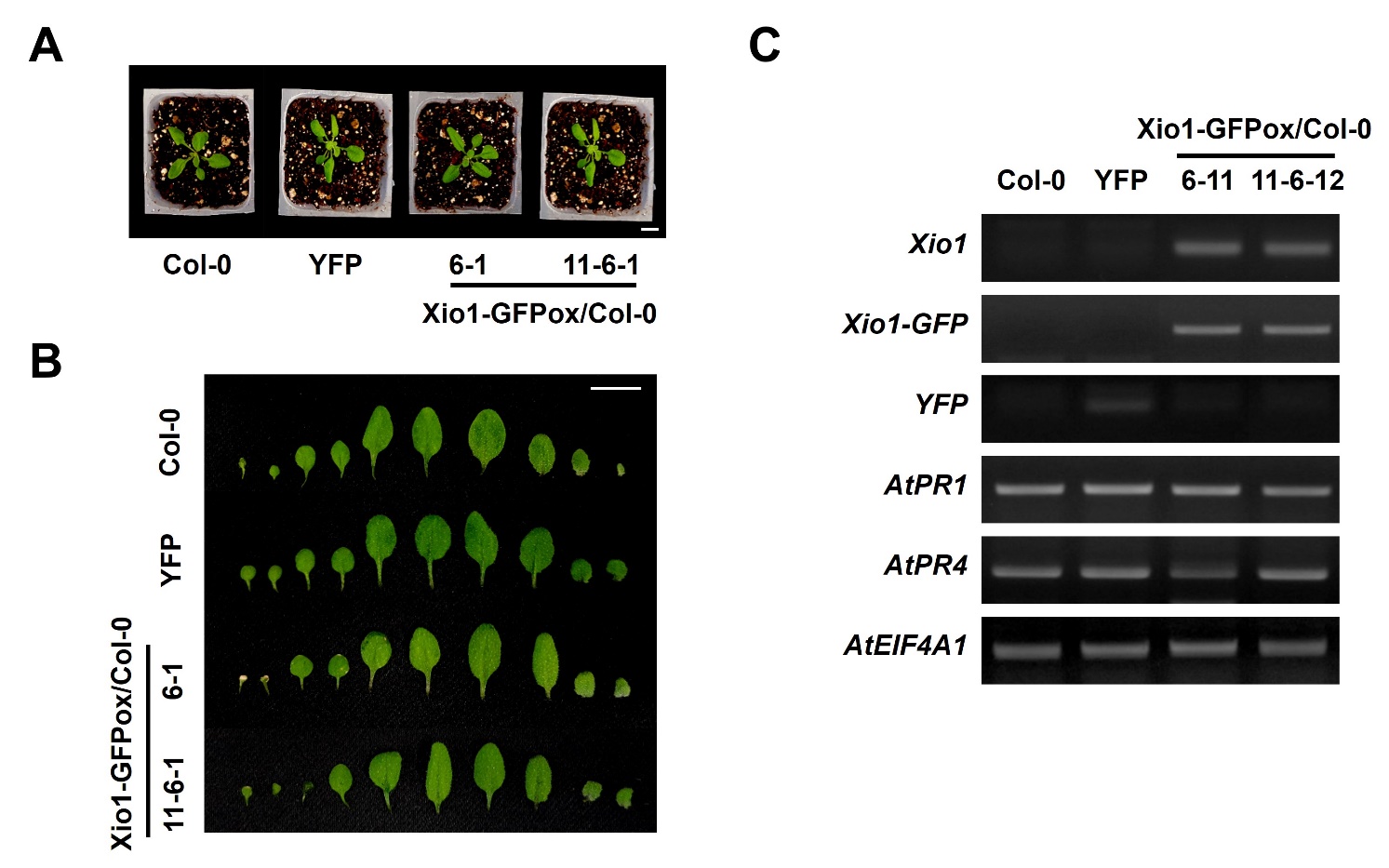
**

**Supplementary Figure 10.** Phenotypic analysis of transgenic Arabidopsis plants overexpressing *Xio1-GFP*. **(A)** Growth comparison of 3-week-old wild-type Arabidopsis (Col-0), transgenic Col-0 lines overexpressing YFP (YFP), and transgenic Col-0 lines overexpressing Xio1-GFP (Xio1-GFPox/Col-0, lines 6-1 and 11-6-1) grown in soil under normal conditions. Scale bars, 1 cm. **(B)** Photograph of individual rosette leaves of Col-0, YFP, and Xio1-GFPox/Col-0 (lines 6-1 and 11-6-1). Leaves are placed from the ﬁrst to the latest leaf in that order. Scale bars, 1 cm. **(C)** Expression analysis of Xio1-GFPox/Col-0 (lines 6-11 and 11-6-12). RT-PCR was performed using specific primers for *Xio1*, *Xio1-GFP*, *YFP*, *AtPR1*, *AtPR4* genes. Eukaryotic translation initiation factor 4A1 (AtEIF4A1) was used as an internal control.
